# Mate choice strategies in a spatially-explicit model environment

**DOI:** 10.1371/journal.pone.0202680

**Published:** 2018-08-23

**Authors:** Giordano B. S. Ferreira, Matthias Scheutz, Sunny K. Boyd

**Affiliations:** 1 Department of Computer Science, Tufts University, Medford, MA, United States of America; 2 Department of Biological Sciences, University of Notre Dame, Notre Dame, IN, United States of America; University of Missouri Columbia, UNITED STATES

## Abstract

Decisions about the choice of a mate can greatly impact both individual fitness and selection processes. We developed a novel agent-based model to investigate two common mate choice rules that may be used by female gray treefrogs (*Hyla versicolor*). In this model environment, female agents using the *minimum-threshold* strategy found higher quality mates and traveled shorter distances on average, compared with female agents using the *best-of-n* strategy. Females using the *minimum-threshold* strategy, however, incur significant lost opportunity costs, depending on the male population quality average. The *best-of-n* strategy leads to significant female:female competition that limits their ability to find high quality mates. Thus, when the sex ratio is 0.8, *best-of-5* and *best-of-2* strategies yield mates of nearly identical quality. Although the distance traveled by females in the mating task varied depending on male spatial distribution in the environment, this did not interact with female choice for the *best-of-n* or *minimum-threshold* strategies. By incorporating empirical data from the frogs in this temporally- and spatially-explicit model, we thus show the emergence of novel interactions of common decision-making rules with realistic environmental variables.

## 1. Introduction

Non-random patterns of mating can have significant consequences for individual fitness and evolutionary processes. For example, in the common case, male mating success is highly variable and dependent on multiple aspects of female choice such that many males may not mate at all in some species [1–3]. Female choice of males may be influenced by a broad range of internal factors (e.g., preferences, decision-making rules, energy reserves) or external factors (e.g., male-male interactions, male spatial distributions, predation risk [4]). Discovering the relative importance of such factors to choice for individual females is a long-standing problem. Here, we investigate possible decision-making rules that females may use to choose mates, using an agent-based model based on frog social behaviors.

Choice of a mate has much in common with other decision-making tasks, such as foraging. Resources are dispersed to varying extents in the environment. Choosers must sample (collect information about resources), rate resources in value based on some preference function or functions, and make decisions that integrate that value ranking with the investment required to procure the resource [4–6]. The distribution of males in the environment is thus also a possible factor in female mate choice (eg, [7, 8]). The male distribution—relative to each individual female—may alter sampling processes of females, preferences, and/or the investment required to exercise a choice. Agent-based models (ABM) are advantageous for problems of this sort because they allow us to examine the choice processes over time and include the explicit representation of the distribution of male and female agents in space.

Mate choice decisions are context dependent and individual decisions may not necessarily contribute to overall lifetime fitness. Organisms can make mistakes and learn to improve their decisions or make apparently irrational or paradoxical decisions [9–11]. Strategies evolved for particular environments may perform poorly in others [8, 12–14]. We implemented a model that did not assume optimality but rather examined the actual performance of realistic strategies in realistic environments. We did not assume every decision on a given night would maximize fitness, given the imperfect knowledge females likely possess about their choices and the long-term consequences of those choices. Rather, we focused on the moment-to-moment decisions females might make, based on two classic theories of mate choice decision-making, and allowed some consequences to emerge from interactions with the environment in order to identify particular strategies that may be advantageous in one context but not another.

Agent-based modeling has become an important tool in disciplines investigating complex systems with many interacting entities [15, 16]. These models view entities in the model domain as individuals or “agents” that can act independently in the “environment” based on their inputs. Such models are increasingly used for understanding and predicting social behavior [17–23]. Models using the agent-based framework are well-suited to integration of information across disciplines and levels of biological organization [24, 25]. The temporally- and spatially-explicit nature of the models captures these two essential features of social interactions.

We investigated two prevalent models of mate choice strategy. In the first case, the *best-of-n* rule (also called fixed search or pooled comparison rule), the choosier sex (assumed to be females here) employs a decision-making rule whereby she assesses a fixed number of possible mates (*n*) and then chooses the one with the highest quality [26]. In the second case, the *minimum-threshold* rule (also called the sequential search rule), a female employs a decision-making rule where she chooses the first mate during a sequential search that exceeds an internal threshold [27]. Both strategies have been subjects of significant theoretical and empirical work (e.g., [28–31]). We have taken the novel approach of using extensive empirical data on treefrogs to test the success of these two theoretical decision-making strategies, including realistic spatial and temporal constraints.

Social aggregations are widespread across animal phyla and can serve a host of functions, most of which are presumed to improve individual fitness [32]. In anuran amphibians (frogs and toads), many species congregate in the hundreds at conspicuous sites ("choruses") where loud vocalizations serve for mate attraction, mate choice, and in male-male competition [14, 33–35]. We modeled the mate choice behaviors of the common North American gray treefrog, *Hyla versicolor*. This frog species relies on vocal communication for social interaction and a wealth of information exists on their social system (e.g., [36]). Anuran amphibians have a long history as model organisms in the study of mate choice by females [14, 37, 38]. The stereotypy in frog behaviors and exclusive dependence of most female choice behavior on auditory communication provide advantages in the creation of realistic but simple models. In addition, due to the conservation of many behaviors across vertebrates, we expect the treefrog model to be broadly applicable to mate choice in other species and to provide a framework for the development of species-specific models.

In this model, male and female treefrog agents interact in a virtual environment where male agents vocalize to advertise their location and quality and female agents choose mates based on their own decision-making rules. In contrast to most prior models, our model focuses on single decisions by females, rather than the evolution of mate choice rules. We thus model the success of strategies over a single night of decision making, rather than over many nights, seasons, or generations. In addition, the performance of different mate choice strategies is usually intimately associated with the costs of searching. In the gray treefrog system, a female can theoretically hear and thus sample many males from a single location so we do not include search costs explicitly. Instead, several types of costs of particular strategies emerge from the model and reflect complex interactions of strategy and environmental variables. The model thus provides new insights into the relative performance of different mate choice strategies for individual females, depending on a set of realistic constraints.

## 2. Model and methods

### 2.1 The biological model system: The gray treefrog, *Hyla versicolor*

Male gray treefrogs arrive at chorus sites (wetland areas) in the early evening and take up residence on ephemeral territories (with distances of 75 cm or more between individuals) from which they advertise vocally [35]. Males often (about 50% of the time) can be found on another site on future nights. On these sites, males defend no resources of obvious utility to females, and oviposition usually occurs in deeper water distant from the males’ calling sites. Mating lasts for several hours and thus males that mate on a given night cannot return to the pool of available males for that night [39]. There is no parental care.

Choruses are active for various lengths of time in spring and early summer, depending on the weather (commonly 5–50 nights per year), with males calling for 3–4 h beginning shortly after dusk [35]. Individual males call for 1–20 nights within a single year’s chorus (mean of 7 nights; [2]). Males arrive asynchronously (over periods of weeks) and may disappear for days or weeks before reappearing to call again during the same season [1, 2].

The species-specific advertisement call of the male attracts females, who approach and contact specific males for mating [35]. Females usually mate only once per season, but enter the chorus asynchronously so the number of females on a given night is variable across the season [2, 39–41]. Specific parameter settings appropriate to this species may be found in Table C in S1 File.

### 2.2 Agent-based model basic design principles

The model is fully described according to the ODD (Overview, Design concepts, Details) protocol [42, 43] in Section A.2 in S1 File.

This agent-based model was designed to understand social interactions of male and female agents in a biologically plausible mating task. In the context of the treefrogs, male agents located in the swamp call to attract female agents and, based on the attractiveness of those calls and the distance to the male, females choose a male to mate. The model applies two particular theories for optimal mate choice strategies—the *best-of-n* strategy or the *minimum-threshold* strategy—to the specific case of gray treefrogs. Our goal was to understand the performance of the two strategies (compared to a control condition with random choice) in a spatially and temporally explicit model environment with realistic variability in potential mate quality, mate locations, and operational sex ratios.

The strategies we investigated represent the two primary general classes for mate choice decision making. Both the *best-of-n* and the *minimum-threshold* strategies can include modifications which may well represent more realistic strategies for particular species. For example, females using the *minimum-threshold* strategies may change their thresholds over time, rather than using the fixed thresholds we implemented [44–46]. We sought to directly compare our model implementation with the classic, non-species-specific models for these strategies [26, 27]. Future plans include the extension of these models to address other potential mate choice strategies.

Female frogs commonly show phonotaxis toward the advertisement calls of conspecific males, when they are searching for mates. Females can clearly discriminate between calls and they show preferences for particular call features. In gray treefrogs, females show enhanced phonotaxis toward calls with more pulses per call (the *pulsenumber*; [47–50]). Importantly, we define “preference” and “choice” *sensu* Jennions and Petrie [4]. “Preference” is the order in which a female ranks prospective mates. “Choice” incorporates these preferences plus the costs. Thus, a female may prefer a male with a high pulse number, but if he is too far away she will choose a closer mate with a lower pulse number. Although there are many factors that may affect a female’s choice, we only consider call quality (as reflected in call *pulsenumber*) and distance in this initial model. We therefore assume females make an active choice [51] and show a directional bias (more pulses are better; [52]). Female anurans are well known to discriminate sharply between vocalizations in the lab and those decisions can influence offspring fitness [36, 53–55].

Auditory sensitivity is sufficient for frogs to theoretically detect the calls of all others in a chorus the size of ours (10 x 25m; [56]). There is evidence that female treefrogs (as well as some other taxa) use simultaneous or “cluster” sampling, where many potential mates can be evaluated from a single location [48, 57–60]. Field data reports female frogs remaining stationary for extended periods before making direct approaches to a single male in a group [1, 3]. Female agents in our model are thus assumed to hear all males in the environment. This can lead females using the *minimum-threshold* or *random* strategies to target males anywhere in the swamp. All females in a single simulation use the same strategy.

On the other hand, it is unlikely female treefrogs can separate all individual calls in a chorus from each other. In natural choruses, noise generated by the calls of conspecifics, as well as males of other species at the same site, clearly interfere with the ability of females to localize individual males [11]. It has been proposed that female frogs only evaluate the nearest 1 to 5 males [11, 61–63]. Empirical data in some frog species also supports the contention that females simultaneously assess a small number of males and make relatively rapid decisions [48, 57, 58]. Thus, we implemented the *best-of-n* strategy by having female agents choose the best male (highest *pulsenumber*) heard from the closest *n* males. We varied *n* from 1 to 5 in increments of 1. We implemented the *minimum-threshold* strategy by having female agents choose the closest male heard whose quality (*pulsenumber*) was above their minimum threshold for acceptance. Lastly, for the *random* strategy, females choose a male at random from any location in the swamp. As in natural choruses, time is limited and mating removes both sexes from the available pool for that night. The choices available to female agents change over time, depending on prior mating and the female agents' spatial position. Females using the *best-of-n* strategy re-evaluate (and perhaps “change their minds”) at each time step as the male agents within the set of *n* closest may change [3, 11]. A new male member of the set may be superior in quality to prior members. On the other hand, females using the *minimum-threshold* or *random* strategies only change their target male if that male disappears due to mating. Females are removed from the simulation by mating or, in the case of the *minimum-threshold* strategy only, when no males above threshold are present in the environment. Because females using the *minimum-threshold* strategy can hear every male in the swamp, if no males above threshold are detected at one time step, then none will ever be detected later.

The model environment was a fixed dimension “swamp” of realistic size (10x25m; no wrap at the edges), applicable across diverse treefrog environments with dry land, bog and open water [1]. Female agents were randomly distributed at the edges at initialization. Male agents were distributed across the swamp according to either a Gaussian (centered on the swamp), inverse Gaussian (with more males on the edges) or random distribution (S2 Fig). Male agents were distributed sequentially such that each male occupied a territory of at least 50 cm diameter [35]. Males have a *pulsenumber* drawn from a distribution with a mean of 6, 12, 18, or 24 pulses per call (with standard deviation of ± 2 pulses) and the *pulsenumber* for a given male does not change. Males do not move but females make a direct approach toward targeted males. The movement rate of females is realistic [64] such that time to reach targets might be protracted and other females may reach the targeted male first. Males are removed from the simulation only by mating. Male and female agents removed are not replaced in the environment. Thus, the quality of mates available changes over time.

The model simulates a single evening of mating activity. In natural choruses, treefrogs congregate at chorus sites in early evening and then males call and females choose mates over about 3–4 hours [65]. We used a realistic simulation cycle time of 1 sec, where agents sense their environment and act on that information in 1 sec time steps. The model thus allowed a maximum time of 14,400 sec. However, in practice, no simulation lasted more than 3,516 cycles, which may reflect male and female agents starting synchronously. In the field, treefrogs arrive at the swamp asynchronously. Thus, time was not limiting in the simulations and no agents had searching or mating behaviors terminated by a time limit. The average cycles per simulation was 673.

### 2.3 Simulation experiments and data analysis

In order to compare the performance of mate choice strategies in the mating task, we systematically varied several parameters. For each point in multidimensional parameter space, we ran 100 simulations with distinct initial conditions. This parameter sweep resulted in 48000 simulations.

We compared agent behaviors across ten different strategy:parameter conditions, based on empirical data for treefrogs (see Section A.2 in S1 File). This included 5 versions of the *best-of-n* strategy (n = 1 to 5 in increments of 1), 4 versions of the *minimum-threshold* strategy (threshold θ = 6, 12, 18, or 24 pulses per call), and a *random* choice strategy. The quality of mates available can influence success of female strategies so we varied the mean pulse number of males across simulations by drawing the male population *pulsenumber* from Gaussian distributions with means of 6, 12, 18 or 24 pulses per call (with σ = 2 in all cases) [50, 66, 67]. In many frog species, the number of males in the chorus on a given night may greatly exceed the number of females [1, 3, 39]. Empirical information for gray treefrogs gives operational sex ratios from 0.04 to 0.82 [63, 65]. In our simulations, we performed a parameter sweep of sex ratios from 0.2 to 0.8. Thus, simulations included between 5 and 20 females (in increments of 5) and a fixed number of 25 males. Finally, we varied the spatial location of males in the swamp according to Gaussian, inverse Gaussian (places more males on the edges, as might occur on pond shorelines), and random distributions (example S2 Fig; references in Table C in S1 File).

In our statistical analysis, we treated each strategy and parameter pair as a unique treatment. Our implementation uses a decision-making framework based on classic biological strategies, such as the *best-of-n*, *minimum-threshold*, or *random* strategies. However, only a specific parameterization of a strategy creates a testable model outcome. Thus, for example, we must fix *n* in *best-of-n* to 1 or 5 (or other) to implement it. In addition, in practical terms, identical outcomes may derive from very different strategies, depending upon their parameterization. For example, the strategy defined by *best-of-1* is the same as "take the closest" for the mating tasks in all environments in our model. Moreover, whether two strategies have the same or different effects might depend on other parameters. For example, *best-of-1* and *minimum-threshold* for θ lower than the lowest quality of any male present in the environment converge to the same strategy. (As an example, if the male agents’ *pulsenumbers* are drawn from a distribution with a mean of 24 pulses per call and SD = 2, then the likelihood of any males with *pulsenumbers* less than 6 in the available population is very small. Therefore, if the *minimum-threshold θ* for female agents is 6, females in this environment will choose the closest male. This results in identical choices by females in the *minimum-threshold* strategy and the *best-of-1* strategy.)

We performed three univariate ANOVA analyses (one for each dependent variable), across parameter sweeps for the four independent variables (see Table 1 and Table 2). This allowed us to examine the outcomes for each of the ten strategy:parameter pairs separately, as well as identify potential interactions with the number of females, male spatial distribution, or male population pulse number variables. When warranted by the ANOVA results, we followed this with Tukey’s HSD post-hoc tests and/or Cohen’s *d* measures of effect size. The statistical analysis was all performed in R (R version 3.3.1; 2016-06-21;[68]).

**Table 1:** Summary of means (± 95% confidence intervals) for each dependent variable across mate choice strategies.

Strategy	Pulse Number of Male Mates	Distance Traveled by Females (cm)	Number of Males Targeted before Mating	Percent of Females that Mate
***Minimum-threshold***	18.5 ± 0.1	287.6 ± 3.7	1.1 ± 0.01	61
***Best-of-n***	15.9 ± 0.1	588.1 ± 3.2	2.3 ± 0.01	100
***Random***	15.0 ± 0.2	1123.6 ± 5.8	1.3 ± 0.01	100

**Table 2:** Effects of female mate choice strategies, sex ratio, and the distribution of male quality and spatial locations on the quality of mates females find, the distance they travel and the number of males they target during the mating task (univariate ANOVA results for each dependent variable).

	*Pulse Number of Male Mates*			*Distance Traveled (cm)*			*Number of Males Targeted*		
Variables	*df*	*F* value	*p*	*df*	*F* value	*p*	*df*	*F* value	*p*
Strategy:parameter (sp)	9	70050.0	*<* .001	9	20945.8	< .001	9	17562.3	*<* .001
Number of females (nf)	3	1803.4	*<* .001	3	7639.4	< .001	3	20639.1	*<* .001
Male spatial distribution (md)	2	49.0	*<* .001	2	1323.5	< .001	2	970.0	*<* .001
Male population pulse number (mp)	3	1633400.0	*<* .001	3	1422.8	< .001	3	3466.7	*<* .001
sp:nf	27	226.4	*<* .001	27	291.3	< .001	27	744.3	*<* .001
sp:md	18	2.4	*<* .001	18	149.2	< .001	18	45.3	*<* .001
sp:mp	24	1229.5	*<* .001	27	1319.8	< .001	27	1993.7	*<* .001
nf:md	6	8.3	*<* .001	6	5.2	< .001	6	73.6	*<* .001
nf:mp	9	0.24	*ns*	9	20.9	< .001	9	54.9	*<* .001
md:mp	6	0.06	*ns*	6	17.8	< .001	6	8.5	*<* .001
sp:nf:md	54	0.61	*ns*	54	2.2	< .001	54	3.7	*<* .001
sp:nf:mp	72	1.07	0.323	81	25.8	< .001	81	45.0	*<* .001
nf:md:mp	18	0.2	*ns*	18	0.1	*ns*	18	0.9	*ns*
sp:nf:md:mp	192	0.8	*ns*	216	1.6	< .001	216	0.8	*ns*

*ns* = not significant

As a measure of fitness of female agents displaying specific strategies, we propose that fitness per night varies linearly and is in part captured by a variable “α.” This variable can represent different environmental pressures (that may differ across nights) and balances the importance of the quality of mates and the distance traveled, the two primary dependent variables we investigated. For example, α close to 1 would represent the case where more weight should be placed on distance traveled, rather than mate quality (perhaps because of predation pressure). On the other hand, a value close to 0 would represent the case where more weight should be placed on mate quality, rather than distance (perhaps due to abundant energy reserves for the female). We used this formula to calculate fitness: Fitness = (1 - α) * ((MeanPN-MinPN)/(MaxPN-MinPN)) + α * (1- ((MeanDist-MinDist)/(MaxDist-MinDist))). [Please note the definitions: MeanPN = Mean pulse number of mated males; MeanDist = Mean distance traveled by females; MaxPN = Maximum pulse number of mated males in the entire database; MinPN = Minimum pulse number of mated males in the entire database; MaxDist = Maximum distance traveled by females in the entire database; MinDist = Minimum distance traveled by females in the entire database. When females did not mate, the quality of their mates was 0].

## 3. Results

### 3.1 Summary

Extensive explorations of parameter space showed significant differences in the performance of the strategies and also the emergence of important costs to females related to travel to mates, female:female competition, and lost opportunities. There were significant main effects of strategies, number of females in the simulation (sex ratio), the male spatial distributions in the environment, and the male population average pulse number on all three dependent variables (Table 1 and Table 2). The *minimum-threshold* strategy, in this model environment, performs better than the other two strategies, with female agents finding better quality mates (with higher *pulsenumber* serving as a proxy for better quality), traveling a shorter distance, and switching targeted males fewer times. On the other hand, a much greater percentage of females find no mates at all when using the *minimum-threshold* strategy due to cases when there are no males whose *pulsenumber* is higher than threshold. All females that use the *best-of-n* or *random* strategy mate in one night. Females using the *best-of-n* strategy target a higher number of mates before mating, due to their reassessment of choices at each time step. Females using the *minimum-threshold* or *random* strategy only reassess if another female mates with their chosen male. Lastly, the distance traveled before mating is greatest for females using the *random* strategy because they can choose from anywhere in the swamp. Females using both other strategies have a restriction that leads to preference for closer males.

### 3.2 Do females find better quality mates with the *best-of-n* strategy or the *minimum-threshold* strategy?

The quality of mates that females find is profoundly influenced not only by the particular strategy, but also by the precise setting of the strategy parameters, *n* or θ. Importantly, this means that some strategy: parameter pairs lead to identical outcomes, even though the conceptual framework behind their implementation is quite different.

**Fig 1:**
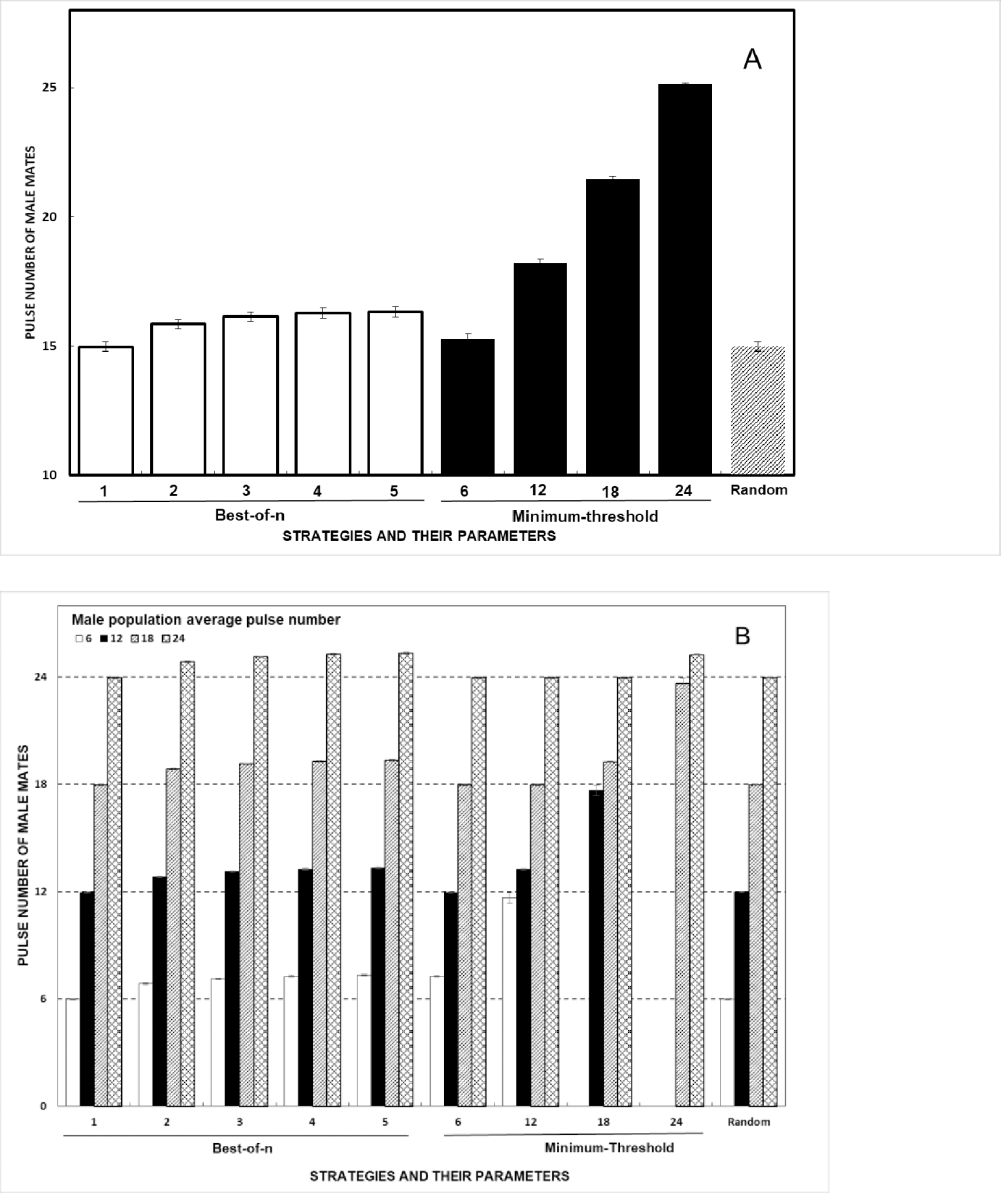
Effects of female agent mate choice strategies on the quality of male mates that females find (mean ± 95% confidence intervals). (A) Results for each strategy:parameter pair. (B) Results presented based on the male population average pulse number. The horizontal dashed lines indicate the four average population pulse numbers investigated.

The significant main effect of strategy on male mate quality (*pulsenumber*) is driven by increasing thresholds in the *minimum-threshold* strategy that limit female choice of lower quality mates (Fig 1A, Table 2). Although the *pulsenumber* of male mates increases significantly for the *best-of-n* strategy as *n* increases, this effect is quite small (Cohen's *d* measures of effect size ≤ 0.2). Not surprisingly, the *random* strategy and the *best-of-1* strategy are not significantly different from each other (Tukey's HSD post-hoc).

### 3.3 Where should females set their *n* or *minimum-threshold θ* in relationship to the male population average in order to find the best mates?

Success of different female mate choice strategies was profoundly affected by the distribution of male quality present in the populations from which females chose. There was a significant main effect of male population average *pulsenumber* on the final quality of mates that females find and a significant interaction of the population average with female strategies (Fig 1B, Table 2).

Under these model conditions, use of the *best-of-n* strategy when *n* >1 results in modest benefits for female agents, in terms of male quality only. For females using the *best-of-2* strategy, the average *pulsenumber* of male mates improves by 6% and *best-of-5* results in an improvement of only 9%, compared to *best-of-1* (less than 2 pulses per call; Fig 1A).

The *minimum-threshold* strategy results in dramatic gains in male mate quality for female agents when thresholds are set high. When the female threshold is set at a *pulsenumber* of 24, the average quality of mates females find is about 10 pulses per call above (63% increase) the quality found with the *random* strategy (Fig 1A). However, there is a complex interaction between the male population average and the female thresholds. First, if both values are the same or θ is lower than the male population average, most or all females find mates but the quality of those mates is not significantly better than the quality found with the *best-of-n* or *random* strategies (about 1 pulse per call difference). Second, when a female agent's threshold θ is 3 standard deviations greater than the male population average (e.g., population average is 6 pulses per call but the female θ is 12), only a few males will have call pulse numbers above threshold. Thus, very few females mate but the benefits to those females are large, compared to the *random* strategy. For example, when the female threshold θ is set to 18 and the male population average is 12, the quality of mates females find is 48% better than with the *random* strategy. Lastly, when θ is more than 3 standard deviations above the male population average (e.g., θ = 24 and male population average = 12), no males usually exist in the simulation with *pulsenumber* above threshold so no mating occurs.

As expected, the *random* and *best-of-1* strategies exactly track the population average. When males of different quality are randomly distributed across the space, whether females chose the closest male (as in *best-of-1*) or a male from anywhere in the swamp (as in *random*), the average *pulsenumber* of male mates is the same.

### 3.4 Which strategy results in the least travel cost to females?

**Fig 2:**
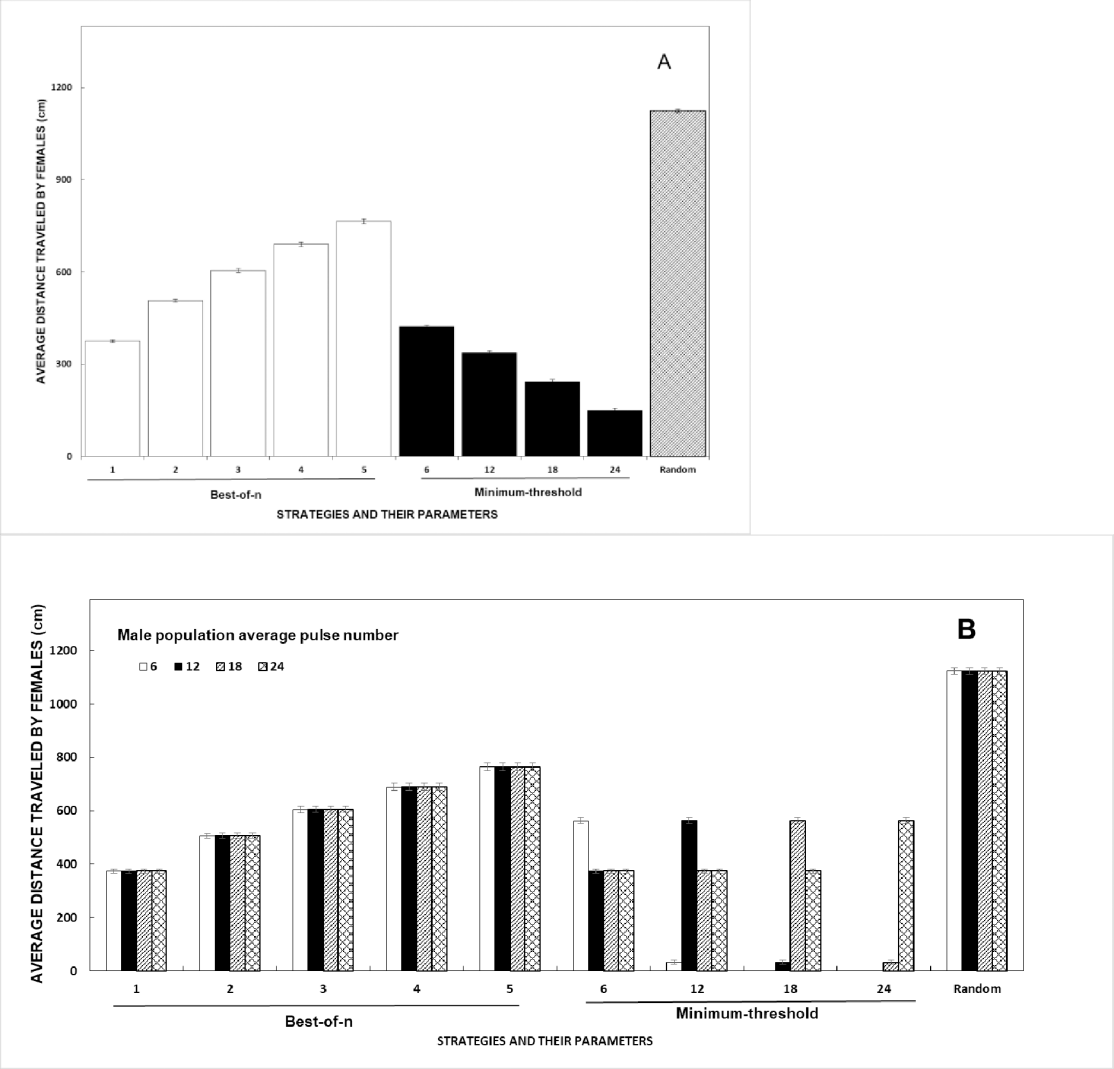
Effects of female choice strategies on the average distance traveled by females to reach mates (with 95% confidence intervals). (A) Results for each strategy:parameter pair. (B) Results presented based on the male population average pulse number.

We did not include search costs in this model (based on the behaviors of real treefrogs) but the distance traveled by females in reaching chosen mates emerged from the model. Although distance traveled to reach mates may not always be a cost, it is likely to be so for female treefrogs [8, 35, 61, 69, 70]. We found interesting interactions between the strategies, how the parameters *n* and θ are set, and the average male population quality. There was a significant main effect of strategy on the distance traveled by female agents engaged in the mating task (Fig 2, Tables 1 and 2). Females using the *random* strategy travel the farthest, as their choices may be located anywhere in the swamp (Fig 2A and 2B). Of course, distance traveled by females using this strategy is not influenced by the male population average quality (Fig 2B).

For females using the *best-of-n* strategy, the distance traveled increases significantly as *n* increases, until females using *best-of-5* travel about twice the distance of those using *best-of-1* (Fig 2A). Note that the shift from considering 1 vs. 5 males confers only a modest (at best) advantage in ultimate male mate quality (Fig 1). The increase in distance traveled as *n* increases is influenced by two factors. First, there is a significant main effect of strategy on the number of males targeted before mating, such that females using *best-of-n* target on average twice as many males, compared to females using *random* or *minimum-threshold* strategies (Tables 1 and 2). Because females using *best-of-n* can reassess males at each time step, they may "change their minds" as new better males appear in the set under consideration. Second, there is a significant interaction between strategy and the number of females in the simulation for distance traveled (Table 2). This manifests as an increase in female:female competition that is only significant for females using the *best-of-n* strategy (section 3.5). This leads to females being forced to choose new mates—and travel toward new destinations—more often at high sex ratios. The average quality of males in the population did not influence the distance traveled by females using the *best-of-n* strategy (Fig 2B).

On the other hand, for females using the *minimum-threshold* strategy, the distance traveled by females significantly decreases as the value of the minimum threshold θ increases (Fig 2A and 2B). Importantly, Fig 2 includes data from those female agents who find mates and those who ultimately do not. As the minimum threshold increases, more (perhaps all) females will find no males above threshold (eg, for θ = 24 and male population average *pulsenumber* of 6, 12, or 18; Fig 2B). In these cases, females perform only a brief random walk for one cycle before being removed from the simulation. This decreases the average distance traveled by all females.

We also considered the distance traveled separately for females that mate and those that ultimately do not (Table A in S1 File). When we include only females who mate, the average distance traveled by females using the *minimum-threshold* strategy is 423 cm, which is still significantly less than the distance traveled by females using the *best-of-n* strategy (588 cm; Table 1). Thus, distance traveled with the *minimum-threshold* strategy is still significantly shorter than for the *best-of-n* or *random* strategies, whether computed for females that mate only or with non-maters averaged in.

### 3.5 Which female mate choice strategy leads to the most female competition?

**Fig 3:**
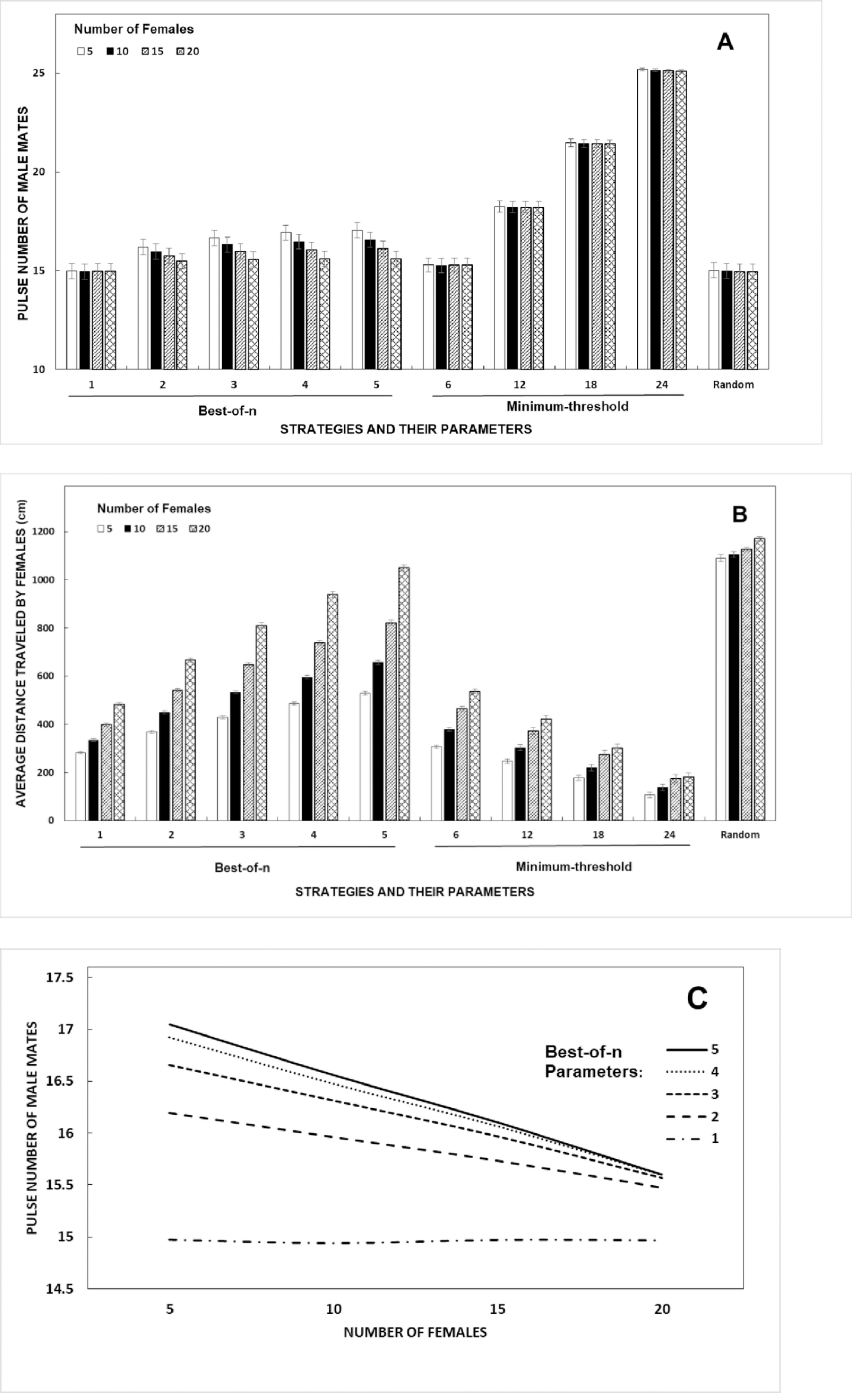
Effect of number of female agents in the simulations (number of males held at 25). (A) Effects on the pulse number of male mates that females find, (B) average distance traveled by females, and (C) comparison of the outcome (final male mate quality) for all 5 *best-of-n* strategies, depending on the number of females. All values for Fig 3A and 3B are means ± 95% confidence intervals. Fig 3C shows means only.

In this model environment, the number of males was held constant at 25 and the number of females per simulation was increased, up to an operational sex ratio of 0.8 (20 females; references in Table C in S1 File). Thus, although there were always more males than females, female mate choice strategies may limit choices nonetheless and lead to females competing with each other for available mates. We found that the success of the *best-of-n* strategy was most affected by changes in the sex ratio, compared to the *minimum-threshold* and *random* strategies. There was a significant main effect of the number of females on the quality of male mates and distance traveled by females, as well as a significant interaction with the specific parameters within each strategy for both dependent variables (Table 2, Fig 3).

For females using the *best-of-n* strategy and where *n* >1, increasing the number of females significantly decreases the average pulse number of male mates that females find (Fig 3A and 3C). As the number of females increases, the probability of 2 or more females pursuing the same male also increases. For the *best-of-n* strategy in particular, because females can "change their minds" when a better male becomes part of the *n*-closest set, this problem is especially acute. The likelihood that two females will have the same “best” choice is higher. The quality of the initial male targeted by females using the *best-of-n* strategy is unaffected by the sex ratio. However, the last male targeted (with whom females will mate) is of lower quality when the number of females competing is greater than 5 and *n* >1 (Fig 3C). For *random* and *minimum-threshold* strategies, on the other hand, females only change targets when an initial target male mates with another female, which is much less common. The sex ratio had no significant effect on the quality of mates females find when using the *minimum-threshold* or *random* strategies (Fig 3A). Therefore, competition with other females was not a significant factor in mate quality for those females using the *minimum-threshold* or *random* strategies, even at relatively high sex ratios.

In the case of distance traveled by female agents, there was a significant increase in distance as the number of females increases, for all strategies (Fig 3B). This was largest for females using the *best-of-n* strategy, with an 89% increase when 20 females were present, compared to 5. The effect was intermediate for females using the *minimum-threshold* strategy at 72% and lowest for females using the *random* strategy at just 7%.

### 3.6 Does the distribution of males in the environment influence mate quality or travel costs?

**Fig 4:**
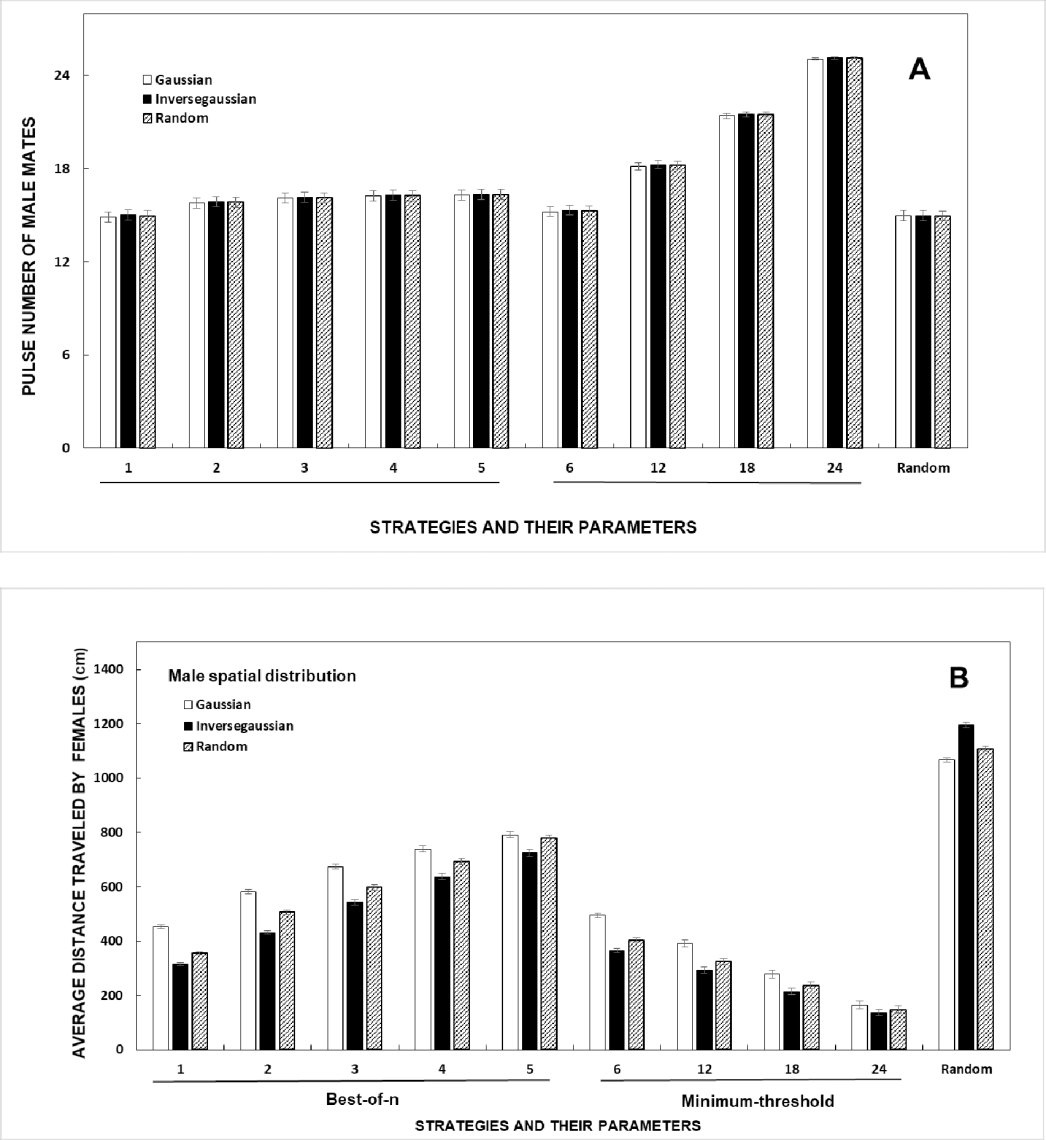
Effect of different spatial arrangements of male agents in the environment on the success of female mate choice strategies (means ± 95% confidence intervals). (A) Pulse number of the male mates that females find. (B) Average distance traveled by females in the mating task.

The spatial distribution of males in the environment may alter female mate choice, especially when females use a decision rule that puts a premium on closest males. There was a significant main effect of spatial distribution of males on the quality (*pulsenumber*) of male mates that females find (Fig 4A, Table 2) and the distance traveled by females in the mating task (Fig 4B, Table 2).

Interestingly, the quality of mates that females find does not differ among different male spatial distributions, within a given strategy and parameter pair (Fig 4A). The distance traveled by females does, not surprisingly, vary with the male distribution (Fig 4B). For the *best-of-n* and *minimum-threshold* strategies, travel distance is shortest when males are arrayed closer to the swamp edges with the inverse Gaussian distribution. The results for the *random* strategy confirm intuition that, in the inverse Gaussian distribution, females choosing randomly across the entire swamp travel farther than when males are distributed randomly or concentrated in the center (Gaussian; Fig 4B).

### 3.7 How is overall female fitness influenced by mate choice strategy?

**Fig 5:**
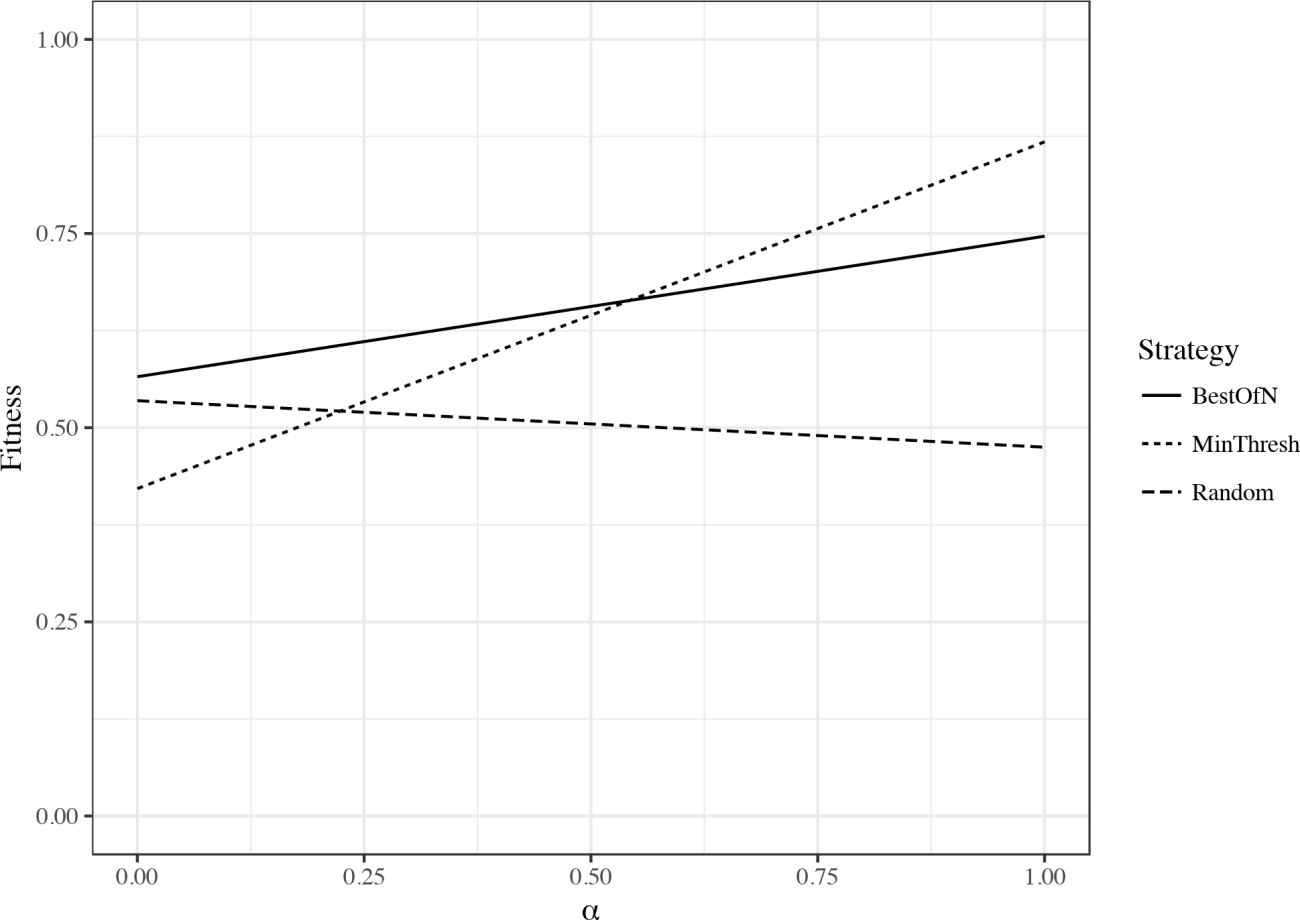
Effect of changes in a variable α on overall fitness of females, depending on mate choice strategy. Fitness incorporates the pulse number of mates that females find (a measure of quality), the distance traveled (a potential cost), and the success of females at finding a mate at all (details in Methods).

The overall performance of any one decision-making strategy can be expected to include a complex interaction among multiple variables. To investigate this aspect of the model, we calculated a “fitness” variable that includes success in the mating task (females that do not mate receive a 0), the quality of male mates (as reflected in male mate pulse number), and the distance traveled by females in the task (as a potential cost). Fitness then depends upon the value of a variable α that can represent different environmental pressures and varies between 0 (when more weight should be placed on mate quality) and 1 (when more weight should be placed on distance traveled as a cost). When α is closer to 1, the *minimum-threshold* strategy performs best and leads to higher fitness values. The *best-of-n* strategy is second best and the *random* strategy performs the worst (Fig 5). On the other hand, when α is closer to 0, the *best-of-n* strategy is best, *random* is second best and the *minimum-threshold* strategy is worst.

## 4. Discussion

### 4.1 Overall strategy comparison

Our agent-based model allowed a novel comparison of mate choice decision rules in the presence of realistic opportunities and constraints. Application of prominent theoretical models to the species-specific situation of gray treefrogs provides a method to assess strategy performance when the model assumptions of neither the original *best-of-n* nor *minimum-threshold* strategies are implemented exactly but rather follow empirical information from the frogs. For example, in gray treefrogs, the aggregation of males into choruses and the acute hearing of females, would theoretically allow females to sample all males from a single location without search costs, so none were explicitly included in our model. In Janetos' influential early model of mate choice, search costs were likewise not included and he found that the *best-of-n* strategy outperformed the *minimum-threshold* strategy, when better performance was based on higher fitness of mates [26]. Real's important foundational model added search costs and found that the *minimum-threshold* strategy outperformed the *best-of-n* strategy under these conditions [27]. When we evaluate strategy performance based on the indicator character of male mate call pulse number (as a proxy for female fitness or male quality; [6] [54]), the *minimum-threshold* strategy was superior despite no search costs. However, this simplification does not consider the significant impact of particular settings of *n* or the threshold θ, the variation in the male quality population average, nor precisely which aspects of the mating task might be deemed "costs."

Implementation of the *best-of-n*, *minimum-threshold* and *random* strategies in this model resulted in some significant changes to the classic models, for fitting to the frog-specific case. First, we have simultaneous sampling possible in all cases. Cluster sampling, as in treefrogs and other leking species, can lead to reassessment of prior males with very low or no costs. Importantly, our model focuses on the decision-making tasks of females on one particular night during a breeding season, in contrast to most prior models which focus on optimal and/or evolutionarily stable strategies. Our consideration of the costs associated with mate choice are thus unique. We can decompose costs into the two general categories of those associated with sampling (or search or assessment) and the costs associated with the consequences of the decisions themselves, such as travel costs to reach a mate or exposure to predators [6, 71]. In lek mating systems used by gray treefrogs, sampling of choices can involve just careful listening from a single location rather than arduous or dangerous travel from male to male as in other species [6, 65]. Thus, our model included no explicit search costs. However, ABM techniques allow easy shifts in the future to accommodate species-specific constraints (e.g., to add search costs, predation pressure, diverse time constraints) depending on individual female physiology or environmental variables.

One can also define optimal strategy performance based on different outcomes of the mating scenario, instead of just final mate quality. For example, optimal performance can mean finding the best possible mate in a single night, if oviposition is imminent. One advantage of our model implementation was that we could observe directly cases when female agents did not mate at all, which sets our model apart from most prior models [6]. This thus represents a "lost opportunity" cost [72]. With our model sex ratio, female agents using *random* or *best-of-n* strategies always mate [6, 65]. At the other extreme, depending on the setting of the female threshold θ relative to the male pulse number population average, we might find that NO female agents find mates in this model. Thus, the lost opportunity costs for females with high thresholds may be very large but are likely modest for those with low thresholds compared to the male population average [8]. Importantly, we model only the female decision making process during a single night's chorus. It could be that an opportunity lost on a single night is not significant (in "real life"). A female frog can return on the next night. The payoff for females with high threshold that return over multiple nights until a suitable high quality male is found may be great. We found the average quality of mates for female agents with the highest thresholds resulted in a 63% increase in quality over random choice. On the other hand, the physiology of ovulation in female frogs means that females move inexorably over a short timespan toward oviposition and those that do not find mates are doomed to lay unfertilized eggs. Females may progressively lower thresholds over a single night or subsequent nights [6, 45, 61]. Whether female frogs return on multiple nights in this species is not clear. In most cases, females are only captured in amplexus and thus at that point oviposition is already impending and mate choice decisions have been made [65, 73]. Tracking small silent female frogs through a chorus at night is a challenge. We consider the number of nights of attendance of female frogs at a chorus to be a key aspect of anuran mating behavior to be explored.

We found that the *minimum-threshold* strategy out-performed both the *best-of-n* and *random* strategies, when male mate quality was the outcome variable considered [20]. Early models assume females "know" the distribution of quality in the population of available males, that the distribution of quality remains constant, and that female thresholds are set optimally in relationship to that distribution [26, 27, 74]. We explicitly examined cases when female thresholds were not equal to the population average. In addition, females remove males from the available pool with mating, so the quality distribution changes over time, usually with best males removed earlier [8, 65]. It is reasonable to expect that the male population average would change over both short and long timescales (e.g., due to stochastic environmental events, directional selection, or predation [8, 12, 13]). For example, significant variability could exist in male quality within a chorusing population [14]. We used a relatively modest and empirically supported constant variation of ± 2 pulses per call [50, 65, 75]. There may thus occur mismatches in female threshold (if genetically determined, dependent on physiological changes that occur over longer time spans, or not learned, among other reasons) and male population average.

### 4.2 Optimal strategy parameters

Female treefrogs in the field may well choose among males with an average pulse number greater than 30, although we did not model such high values explicitly [54]. (Due to drawing from a normal distribution, however, such males did rarely exist in some simulations.) Whether female treefrogs use the *minimum-threshold* strategy—and where the threshold parameter θ might be set if they do—is not known. In lab experiments, about 85% of females prefer a 22 pulse call over 18, however the strength of this preference for long over average calls is not as strong as the preference for average over short calls [50]. This may also represent females using a *best-of-2* strategy and/or a mate choice scenario in the lab with restricted choices, distance, predation risk, etc. A preference for extra-long calls may well have negative consequences for females, however it is reasonable to suggest that female thresholds should be within the highest quartile [33, 50, 72].

In our model, we found only modest benefits for the *best-of-n* strategy over the *random* strategy, in terms of male mate quality, and likewise few benefits for females to increasing *n*. First, we found that moving from *best-of-1* to *best-of-5* results in an improvement in male mate quality of less than 2 pulses per call. Only differences on the order of 10 or 15 pulses have been shown to influence offspring fitness in this species, although even very small differences can influence selection over time [54]. Female treefrogs can discriminate on such a fine scale and, for example, prefer a call with 11 pulses over one with 10 [50]. While a *best-of-n* strategy where *n* = entire population clearly can be an exceptional evolutionary strategy, it is unlikely frogs use this [6]. Female frogs have been shown to attend to 5 or fewer conspecifics at a time [8, 61, 76–78]. This number is typical of other vertebrates, although some instances of females assessing more than 70 males are reported and empirical data implies that *n* varies widely within and across individuals [76, 79]. In our model, we investigated what would happen when females use a *best-of-25* strategy. The same 20 males mate but the approach and duration until everybody mates was very different. Most females pursue the top male, then the second best, and so forth until the last male mates [80, 81]. The *minimum-threshold* strategy, on the other hand, leads to a more parallel search and mating process.

The *best-of-n* strategy is more robust to environmental variation, compared to *minimum-threshold*, because females always mate and there is no need to match threshold θ to the male mean population average [30]. *Best-of-n* may be favored when the ability to locate individuals broadly across the landscape is compromised (e.g., very large choruses when females acquire imperfect information and/or where potential mates move frequently) or when oviposition is imminent and time is of the essence. One can argue that the behavior of male gray treefrogs in a chorus supports the use of a *best-of-n* strategy by females [77]. As chorus size changes in *H*. *versicolor*, male call duration also changes (shorter with fewer male neighbors and longer with more). However the relative rank of males does NOT change. Thus, the male giving the longest, most desirable call in choruses of n = 8 still gives the longest call in a chorus of n = 2, although that call may fall from pulse number of 20 to 12. This implies males seek to be the "best of the set" rather than hitting some potentially high threshold (of which they are capable). Interestingly, one can also consider the use of an alternative *best-of-n* strategy where *n* changes according to environmental circumstances [82]. For example, when males are clumped in patches, *n* could refer to the number of patches in the set rather than the number of males [1, 29, 58, 73].

### 4.3 Sampling and update rules

Many theoretical models assume females do not reencounter males [83]. However, due to cluster sampling by audition in female frogs, resampling is an important component of our model. It is likely that female frogs can update their knowledge of male stimuli rapidly as they move through the chorus, as in our *best-of-n* implementation [3, 11, 58]. Male treefrogs, for example, can change decisions in about 90 seconds about other males with whom they will coordinate calling [61]. This implies that females may likewise be able to detect callers and make decisions on the same timescales. This is especially important as males change position or disappear entirely due to mating [1, 65]. The lek mating system of gray treefrogs thus requires a unique implementation of the reassessment of choices.

In our model, female competition emerged as a significant factor for female agents using the *best-of-n* strategy but not those using the *minimum-threshold* or *random* strategies. The negative effect of female competition in the *best-of-n* strategy was especially profound for *best-of-5* where females traveled 90% farther when the sex ratio was 0.8 compared to 0.2. Because female agents using the *best-of-n* strategy are present at the chorus by definition only ONE night in our model, their choices are greatly affected by the removal of high quality males by female competitors. Under these conditions, the pool of available males changes rapidly and realistically on a single night [8]. Although females using the *minimum-threshold* strategy face competition for rare males above threshold in some cases, in general female:female competition was not an impediment to females using this strategy.

There were two distinct moments in this model implementation when a female agent can change her target male: when the targeted male agent mates with another female or when a better male became one of the *n* closest males in the *best-of-n* strategy. When a female changes target due to a male disappearing with mating, nothing can be deduced about the next target male (i.e., the next target could have a higher or a lower pulse number than the last target due to the random distribution of male quality through the environment). On the other hand, in the case of the *best-of-n* strategy and the appearance of a better male in the set, a change of target will only lead to a better male. However, although females using *best-of-n* can only target males of higher quality at each time step, ultimately this strategy leads to worse quality mates. The first male a female targets with the *best-of-n* strategy had an average pulse number of 16.4 but the last target was 15.96. In the case of *random* and *minimum-threshold* strategies, there was scant difference between the first male a female targeted and the last (for *random*, first target was 14.96 while the last was 14.98; for *minimum-threshold*, first target was 11.83 while the last was 11.84 pulses/call). (The pulse number of the last targeted males noted just above is the average for all female agents including those that ultimately don't find mates and thus differs from Table 1). Therefore, the continuous update by females using the *best-of-n* strategy was harmful to overall performance. Females changing their target male for a better male increased female:female competition. The females tended to pursue the same male (the best local male) and after one female mates, the remaining males are not as good, which makes the next choice a detrimental one.

To further explore the influence of the update rule in the *best-of-n* strategy, we modified the original *best-of-n* strategy, removing the ability to change the target male if a better male becomes one of the *n* closest. Thus, a new male was targeted only if the last target male mated with another female. This modified strategy reduced female:female competition because two females move toward the same male only if both females are initially placed near each other (i.e., both have the same target male). Effects of this change in the model were modest. While the original *best-of-n* strategy resulted in 2.3 different males targeted by females on average, the modified strategy had 1.9 males targeted. For average distance traveled by females, the original *best-of-n* strategy was 588 cm, while the modified strategy was 533 cm. The quality of the mates, measured by the average pulse number of mated males, decreased slightly. For the original *best-of-n* strategy, the average pulse number of the male mates female agents found was 15.9 pulses/call, while the modified strategy was 15.8 pulses/call. When female agents using the *best-of-n* strategy do NOT update at each time step, they develop a common pattern of searching. A group of females move to the position of a local best male and after that, all females will target males in the same order, because they will sample at nearly the same position. Thus, they start to move as a block toward a local best until all females mate. This group searching behavior was worse than changing the target male when a better male becomes part of the closest set. Although small when considered for individual fitness, such differences can have significant effects on an evolutionary time scale. Thus, the original *best-of-n* strategy, with resampling at each time step, was superior from this perspective.

There is limited direct evidence for use of either *best-of-n* or *minimum-threshold* mate choice strategies in frogs, although many such examples are found in other vertebrate classes [4]. Laboratory tests of female treefrog preferences are rarely designed to answer this question, most often using only one or two stimuli [50, 84]. Thus, at best, one might be able to discern a difference between a *minimum-threshold* and a *best-of-2* strategy. We are not aware of studies that directly support use of the *best-of-n* strategy (vs. *minimum-threshold*) by female frogs. In male frog behavior, there is evidence for use of both strategies in the same task [61]. Male tungara frogs seek to space their calls relative to neighbors, such that they lead rather than follow competitors. Arrays of speakers can thus examine to which conspecific calls males respond. This might be considered the same questions that females must answer—(1) "To which male calls should I attend?" (2) "Who is a neighbor?" (e.g., the "closest") (3) "Who is the best competitor/potential mate?" Males under these conditions respond flexibly by changing the number of neighbors to which they respond, with some neighbors falling below an apparent loudness threshold and dropping from consideration while others remain part of a set in which males attempt to be first. This ability to switch strategies is increasingly supported in multiple empirical systems [72, 85, 86] and in models that incorporate modifications such as Bayesian decision making or "last chance" options [6, 8]. Importantly, individual females may use different strategies and/or strategy parameters *n* or θ, so combining field or lab data from many female frogs may not be instructive [4]. We suggest that ABMs may be ideal for defining stimulus parameters that may unambiguously differentiate female mate choice strategies in the lab.

### 4.4 Strategy costs

Our model allows several important costs to females to emerge from agent interactions. These include costs of lost opportunities to mate, travel costs for females to reach male mates, and female:female competition. We found that female agents that choose randomly from the whole chorus travel about 2 to 4 times farther than females that use the *best-of-n* or *minimum-threshold* strategies, respectively. The use of a "closest rule" thus seems favorable even when females choose randomly. Potential mates located farther away represent more costly choices. Such costs are not just restricted to caloric requirements but time spent traveling increases likelihood of predation or oviposition without a mate. In the lab, given a 50% difference in pulse number, the lower quality call must be ~5 dB louder to change female preferences [50]. This suggests female treefrogs put a high priority on distance of potential mates.

In frogs, call amplitude is a critical factor in mate choice and selective attention in males and females and is directly correlated with distance [50, 87]. The inclusion of female agents that find no males above threshold and thus never move beyond the first time step does decrease AVERAGE distance traveled for the *minimum-threshold* strategy (e.g., Table 1). However, even when we consider only the distance travelled by females that ultimately mate (rather than "give up"), the females that use the *minimum-threshold* strategy travel significantly shorter distances. This would likely result in significant savings of energy [69]. We suggest that the use of more realistic distances for mate choice in studies of female phonotaxis may be informative.

Although the *best-of-n*, *minimum-threshold* and *random* strategies appear to us to be very different and this has provided the primary theoretical framework for models and empirical work in the field, our study highlights the importance of considering details of the species-specific implementation of these strategy rules [8]. We found that even females following different strategy "rules" may nonetheless choose the same males for mates. For example, we found that whether females (1) choose randomly, (2) use the *best-of-1* strategy; or (3) use the *minimum-threshold* strategy with theta θ lower than the male quality population average, this results in choice of males with no significant variation in pulse number. Thus, careful attention to both the strategy and parameterization relative to the male quality population average is key. This supports the contention that empirical studies must be carefully designed to differentiate decision-making strategies under realistic conditions. We suggest that ABM might provide a useful experimental test bed to design field and laboratory studies.

In our case of a single night's decisions, *random* choice may have few negative consequences. We found that use of the *best-of-n* strategy instead would result in an average of only ~1 pulse/call advantage. Although females can discern such small differences, it is unlikely this would confer adaptive advantage [54]. Although use of the *minimum-threshold* strategy may confer an average gain of 3.5 pulses/call, females using this strategy may miss opportunities to mate on a given night. Depending on the timing of oviposition, this delay could be catastrophic and result in loss of reproduction for a whole year. This suggests that the cognitive requirements of the *best-of-n* strategy or the lost opportunity costs of the *minimum-threshold* strategy may not be worth the investment in female treefrogs.

### 4.5 Male spatial distributions

Males may not only vary in their quality but also in their spatial distribution such that for females to exercise their preferences, more energy or predation risk or other costs are required to reach desirable males [8]. For male treefrogs, the amplitude of neighbor calls is paramount for determining the distance from competitors, as amplitude varies predictably with distance [35, 61]. Likewise, in female frogs there is evidence that the distance of males is critical [70]. Female frogs discriminate strongly based on the spatial location of males, with strong preferences for closer males. However, under our model conditions, the spatial distribution of males had no significant effects on mate quality although it did, of course, on distance traveled. We distributed males of different quality randomly across the landscape, depending on the desired spatial pattern of the simulation (random, Gaussian, inverse Gaussian). However, leks can have very non-random distributions of males, including high quality "hot shot" males in the center, or "miniclusters" of treefrogs with a calling male surrounded by a few lower quality non-calling satellite males [60, 88, 89]. Under these conditions, *minimum-threshold* or *best-of-n* strategies may perform quite differently, which we plan to address in future work.

### 4.6 Conclusions

In summary, our model presents a unique implementation of classic models of mate choice by use of the ABM framework and the decision-making tasks of female gray treefrogs on a single night of the breeding season. Importantly, this allows us to investigate the importance of known opportunities and constraints, such as the opportunities of the low cost method of "cluster sampling" or the constraints on *n* for the *best-of-n* strategy. Our method also allows us to investigate the realistic situations where neither time nor the pool of available mates are unlimited. Our results suggest that females that switch strategies and/or populations where females may use mixed strategies may be optimal, scenarios we plan to address in future studies.

Our model also provides a readily modifiable framework for species-specific implementation and discovery across diverse taxa (java code included in Section A.5 in S1 File). Straightforward model changes to agent size, quality values, and distributions (for example) allow for investigation of these mate choice strategies across many vertebrate and invertebrate species. We suggest that the use of the model for design of experiments to test mate choice strategies in the field may be a particularly rich arena. Such models may facilitate "dry runs" of experimental studies with animal agents and species-specific stimuli, such that design of field and lab studies can more readily differentiate the strategies used by females [6].

## Supporting information

S1 File Appendix: Supporting information, model description and code. (Table A) Summary of the effects of female agent mate choice strategies across 3 different conditions for female updating of male agent locations (means ± 95% confidence intervals). (Table B) Analysis of the distance traveled by females using the minimum-threshold strategy, divided by whether or not they mate (means ± 95% confidence intervals). (Table C) Descriptions of model parameters for the treefrog mate choice simulation.
(DOCX)

S1 Fig Pseudo-code for the treefrog mate choice simulation algorithm. (TIF)

S2 Fig Representative examples of the initial placements of male and female agents in the swamp environment, according to the 3 spatial distribution types. Locations of males are shown in blue and females in red. The dot size is not to scale but has been increased for visualization purposes.
(TIF)
