# Supplementary material for: Mate choice strategies in a spatially-explicit model environment
Source: PLoS One. 2018 Aug 23;13(8):e0202680. doi: 10.1371/journal.pone.0202680 (PMC6107201; doi:10.1371/journal.pone.0202680)
Supplement: S1 File — (Table A) Summary of the effects of female agent mate choice strategies across 3 different conditions for female updating of male agent locations (means ± 95% confidence intervals). (Table B) Analysis of the distance traveled by females using the minimum-threshold strategy, divided by whether or not they mate (means ± 95% confidence intervals). (Table C) Descriptions of model parameters for the treefrog mate choice simulation. (DOCX) [file pone.0202680.s001.docx]

**S1 File. Appendix: Supporting information, model description and code.** (Table A) Summary of the effects of female agent mate choice strategies across 3 different conditions for female updating of male agent locations (means ± 95% confidence intervals). (Table B) Analysis of the distance traveled by females using the minimum-threshold strategy, divided by whether or not they mate (means ± 95% confidence intervals). (Table C) Descriptions of model parameters for the treefrog mate choice simulation.

**A.1 Supporting information**

Our primary model uses implementations with empirical support from female treefrogs (Table C in S1 File). To summarize, all females sense the location and pulse number of all males at each 1 sec time step. Females using the *min-threshold* and *random* strategy only change their target male if that male is removed from the simulation by mating. The rationale is thus: For females using *min-threshold*, the closest male above threshold remains that unless he disappears. For females using the *random* strategy, a new random choice at each time step would lead to females that never mate (except by accidently bumping into a male). So, females using either *min-threshold* or *random* strategies only update their targeted male if that male is removed. On the other hand, for females using the *best-of-n* strategy, the set of *n* closes males may change at each time step. Thus, females using the *best-of-n* may “change their minds” as they move through the landscape. The results for these implementations is shown in Table 1 and repeated here in Table A in S1 File for comparison with two additional update rules, described next.

For a direct comparison of the three strategies, we also investigated the case where female agents using the *best-of-n* strategy do not update at each time step but instead only change targeted males when the original target is removed by mating (Table A in S1 File). As expected, this leads to no change in any variable for females using the *min-threshold* and *random* strategies. However, females using the *best-of-n* strategy change their targeted male slightly fewer times and travel shorter distances (~55 cm less). Importantly, there is scant change in the quality of mates females find, whether they update at every step or not when using the *best-of-n* strategy.

Next, we considered the case where no female agents update as they travel through the environment (Table A in S1 File). Instead, those using any strategy target one male at initialization and then make a direct approach to that male’s location. When females arrive, they only choose a new target if that male is gone. This means that males can be removed by mating and no longer available long before a female even arrives. As expected, this update rule results in greater distances traveled for all three strategies, although the relative distances for each strategy remain the same. There are no differences in mate quality with this update rule, whether compared to the original model or the one without updates for the *best-of-n*. Likewise, the number of males targeted is the same, compared to the case where all strategies only update when a male is removed by mating.

Lastly, we considered the distance traveled by females in the *minimum-threshold* condition, depending on whether they mated or did not (Table B in S1 File). (Females in the *best-of-n* or *random* conditions always mate.) For females that mate, the distance traveled increases as the *minimum-threshold* increases, as eligible males above threshold will be more distant and rare in the environment. For females that do not mate, the distance traveled decreases as the *minimum-threshold* increases. This is due to an increase in the number of females that hear no eligible males at the first time step and then are removed immediately from the simulation.

**A.2 ODD description of the model**

In this section, we present the model description following the ODD (Overview, Design concepts, Details) protocol [1, 2]. (Parameters are described in Table C in S1 File and model pseudo-code is in S1 Fig.) The model was implemented in our agent-based simulation environment *SimWorld* [3].

**A.2.a Purpose**

The purpose of the model was to examine the interaction between decision-making strategies and environmental variables for female treefrog agents engaged in mate choice tasks. Environmental variables include the operational sex ratio, variability in male quality, and different male agent spatial distributions.

**A.2.b Entities, state variables and scales**

The model has just one type of agent -- the treefrogs. Each agent has some attributes that characterize it at a given time. Specifically, each agent *i* is defined by an unique identity number *i_id_* , its sex *i_sex_*, its location in the environment 〈*i_x_, i_y_*〉 and its size *i_size_*. In addition, female and male agents have some distinct attributes. A female agent has a velocity of movement through the environment *i_v_*, an orientation *i_α_* and a mating strategy *i_π(p)_* . The mating strategy π may be either the *min-threshold* or *best-of-n* strategy, with the strategy parameter *p* setting of either θ (for the specific minimum threshold) or *n* (for the specific value of *n*), respectively. We compared these mate choice strategies with the female *random* choice strategy. On the other hand, a male agent has an advertisement call pulse number, *i_pulsenumber_*.

The agents move in a two-dimensional environment (termed a “swamp”) which has dimensions equal to *E_x_* and *E_y_* . A varied quantity of females are positioned initially randomly (according to parameter *f_δ_* ) on the borders of the swamp to vary the operational sex ratio. Each male (always 25) is positioned inside the swamp in a territory of radius *r_τ_* and according to a particular distribution *m_δ_* (either random, Gaussian, or inverse Gaussian; S2 Fig). Male territories cannot overlap. The pulse number for individual males in a population is based on *µ_pulsenumber_* with standard deviation of σ*_pulsenumber_.* A female agent *i* and a male agent *j* will always mate when they are within mating distance *d_mate_*, i.e., when *d_(i, j)_* ≤ *d_mate_*. Agents that mate are removed from the simulation.

The spatial scale of the swamp is realistic for a gray treefrog chorus in nature at 10 x 25 m. The model runs as a discrete-time simulation where each update cycle corresponds to one second in real-time and the simulation was executed for 60 minutes (3600 cycles), unless termination conditions were reached sooner.

**A.2.c Process overview and scheduling**

The model's primary controller object is responsible for setting the global parameters of the simulation and then creating the agents using those parameters. The controller creates two lists (female agents, male agents) and a hash table with all agents of both sexes. Sequentially, the main loop of the simulation contains the processes performed by the agents until the controller finds a termination condition (S1 Fig). Finally, the dependent variables are calculated in the method *set-Post-Simulation-State-Variables*, and then printed.

Female agents in the model follow the overall sequence of sampling, selecting and approaching hypothesized to occur in female frogs [4]. Male agents just call to attract females and mate if a female comes within mating distance. Processes occur in the following order: (1) female agents sample the available males and target one (*female-sensing*), (2) all agents act differently according to sex. Female agents approach the targeted male (*female-acting*), and male agents determine if a female has approached to a distance closer than *d_mate_* and, if so, then both agents mate (*male-acting*).

The order in which females sense the environment is the same order in which females were added in the list. The acting processes are dependent on the order returned by the hash table containing all agents, thus, depending on the number of females, some of them can be updated before some males and some males can be updated before some females. State variables are updated as soon as they are calculated. However, agents only act after all females have already sensed the environment. All agents therefore act synchronously.

**A.2.d Design concepts**

*Basic Principles.* This agent-based model was designed to understand social interactions of male and female agents in a biologically plausible mating task. In the context of the treefrogs, males located in the swamp call to attract females and, based on the attractiveness of those calls and the distance to the male, females choose a male to mate. The model seeks to apply two particular theories for optimal mate choice strategies -- the *best-of-n* strategy or the *minimum-threshold* strategy -- to the specific case of gray treefrogs. Our goal is to understand the performance of the two strategies in a spatially and temporally explicit model environment with realistic variability in potential mate quality, mate locations, and operational sex ratios.

Female frogs commonly show phonotaxis toward the advertisement calls of conspecific males, when they are searching for mates. Females can clearly discriminate between calls and they show preferences for particular call features. In gray treefrogs, females show enhanced phonotaxis toward calls with more pulses per call (the *pulsenumber*; [5-8]). Importantly, we define “preference” and “choice” *sensu* Jennions and Petrie [9]. “Preference” is the order in which a female ranks prospective mates. “Choice” incorporates these preferences plus the costs. Thus, a female may prefer a male with a high pulse number, but if he is too far away she will choose a closer mate with a lower pulse number. Although there are many factors that may affect a female’s choice, we only consider call quality (as reflected in call *pulsenumber*) and distance in this initial model. We therefore assume females make an active choice [4] and show a directional bias (more pulses are better; [10]). Female anurans are well known to discriminate sharply between vocalizations in the lab and those decisions can influence offspring fitness [11-14]. In some cases, empirical data for *H. versicolor* has not been adequate so we have used data from the diploid sister species of gray treefrog, *H. chrysoscelis*.

Auditory sensitivity is sufficient for frogs to theoretically detect the calls of all others in a chorus the size of ours [15], but it is unlikely they can separate all individual calls. In natural choruses, noise generated by the calls of conspecifics, as well as males of other species at the same site, clearly interfere with the ability of females to localize individual males. It has been proposed that female frogs only evaluate the nearest 1 to 5 males [16-18]. Empirical data in frogs also supports the contention that females simultaneously assess a small number of males and make relatively rapid decisions [6, 19, 20]. Thus, we implemented the *best-of-n* strategy by having female agents target the best male (highest *pulsenumber*) heard from the closest *n* males. We varied *n* from 1 to 5 in increments of 1. We implemented the *min-threshold* strategy by having female agents target the closest male heard whose quality (*pulsenumber*) was above their minimum threshold for acceptance. Lastly, for the *random* strategy, females target a male at random from any location in the swamp.

For all three strategies, female agents make a direct approach to the targeted male, at a constant velocity. At each update, females check that the target male is still calling and has not mated and been removed from the simulation. If the male has been removed, females re-evaluate the closest males at their new position within the simulated swamp. Females using the *best-of-n* strategy re-evaluate the *n* closest males at each time step and may "change their minds" if a better male is now detected from their new location.

*Emergence.* The final mate quality, distance traveled by female agents, number of potential mates targeted, and the likelihood of mating for males and females emerge from the interaction of female choice strategies with the environment.

*Adaptation.* Some agent variables are fixed within simulation, such as the *pulsenumber* of males and the strategy and strategy parameters of individual females. However, females adapt to changes in the environment when target males mate with other females. In this case, female agents target a new male, based upon their strategy, strategy parameter, and their new location in the environment.

*Objectives.* The objective of female agents is to target the best quality mate, according to her strategy choice rules, from a set of closest males. Mate quality was modeled using only the call *pulsenumber* and not incorporating other features of the treefrog advertisement calls. The pulse number of the calls of male *H. versicolor* has been directly linked to the fitness of the females’ offspring, where fathers with a higher pulse number sire offspring with enhanced growth in some environments [12, 21].

*Sensing.* At each update, female agents sense the location and the *pulsenumber* of all males still calling in the environment (those not removed due to prior mating). They use this information to either continue toward a previously targeted male or target a new male.

*Interaction.* Direct social interactions between agents occur only when a male and female agent mate. When agents are within approximately one body length of each other (*d_mate_* ≤ 4 cm), they mate and both are removed from the simulation. Female agents also interact indirectly when they both target the same male and thus compete, with the first female to arrive winning.

*Stochasticity.* Positioning of agents and assignment of *pulsenumbers* to males are each modeled as stochastic processes. At initialization, agents are positioned according to the distributions *f_δ_* for female agents and *m_δ_* for male agents (S2 Fig). The distribution *f_δ_* is random along the edges of the swamp. On the other hand, *m_δ_* can assume one of three distinct distributions: Gaussian, inverse Gaussian, or random. The Gaussian distribution, with means *µ_x_* = *E_x_/2* and *µ_y_* = *E_y_/2* and standard deviations *σ_x_* = *E_x_/4* and *σ_y_* = *E_y_/4*, respectively, results in a greater density of males in the center of the swamp. On the other hand, the inverse Gaussian distribution with means *µ_x_* = 0 or *µ_x_* = *E_x_* and *µ_y_* = 0 or *µ_y_* = *E_y_*, and standard deviations *σ_x_ = E_x_/4* and *σ_y_ = E_y_/4*, produces a higher density of males near the borders of the swamp. Finally, the random distribution just places the males randomly through the entire swamp. Males are assigned sites sequentially, such that if a subsequent male’s territory would overlap with a prior one, a new site is chosen.

Pulse numbers of individual male agents are assigned by sampling a Gaussian distribution with a mean of *µ_pulsenumber_* (with a constant standard deviation of *σ_pulsenumber_* = 2). The population mean is varied across simulations from 6 to 24 in increments of 6. Male agent *pulsenumbers* are rounded to the nearest integer which, in some cases, results in unequal distribution of male quality about the mean.

*Observation.* As this model focused on the mate choice decisions of females, we collected the following data: (1) the quality of mates that females acquired (as reflected in male call *pulsenumber*), (2) the distance traveled by females before mating, (3) the number of males targeted before females eventually find a mate, and (4) the number of females that mate.

**A.2.e Initialization**

*Spatial aspects of the model environment.* The simulation runs in a spatially-explicit "swamp" with fixed dimensions defined as *E_x_* = 10m and *E_y_* = 25m. Male treefrogs usually call from vegetation, the ground, or the water near or in ponds. The size of these ponds can vary greatly depending upon weather. Pond sizes at the beginning of the breeding season were 25 m (in at least one dimension) or more in several published studies [22-24]. We thus choose 25 m as a conservative estimate for one dimension of our simulated swamp. The choice of 10 m for the other dimension resulted in a swamp area of 250 meters (typical of field reports; [23, 25, 26]) and a rectangular chorus site (also typical for frogs arranged along a shoreline). Our model does not include landscape features such as shores or vegetation, so it applies equally to populations of frogs which may call from boggy sites with no defined pond edge (personal observation), those which call from the water only [27], or sites with frogs calling from land and water. For simplicity, we did not model the third dimension (height of frogs above the ground or water surface).

Gray treefrogs are rather large members of the family Hylidae. Empirical data suggest size is unlikely to be a significant factor in mate choice in this species ([22, 23, 28, 29]; but see [30]). Thus, male size was fixed at 4.72 cm [24] and female size at 5.28 cm [31].

*Male agents*. A fixed number of males are placed in the swamp according to a random, Gaussian, or inverse Gaussian distribution, *m_δ,_* and assigned a call with a fixed pulse number according to μ*_pulsenumber_*. Males are placed sequentially in non-overlapping territories (with a radius of 50 cm each; [24]). They continue to call and do not move, unless they mate, in which case they are removed from the simulation. There are neither satellite males nor male-male interactions in this model.

The density of males in the simulated swamp was fixed at 10 males per 100 m^2^ (based on minimum reported by [26]). This resulted in 25 males in all simulations. In the field, the number of males in a chorus on a given night can vary greatly, from 1 to 36 being reported [22, 23]. The total number of chorusing males in our simulation is thus reasonable, calling territories were not limiting, and the density of males is at the lower end of the range, both for *H. versicolor* and for many frog species [24].

*Female agents.* A varied number of females (*f*) are placed at random locations along the swamp edges. All the females in a given simulation have the same strategy *π* and strategy parameter *p.*

**A.2.f Input data**

The model does not use input data to represent time-varying processes.

**A.2.g Submodels**

*Set-Parameters.* This method, the first one called by the main controller, stores the parameters passed to the simulation as instance variables in order to access them by all the other submodels.

*Create-Agents.* The main controller of the simulation creates three data structures to store the agents that exist in the simulation. The data structures are a list with all female agents, *femaleList,* a list with all male agents, *maleList,* and a hash table, *agentList*, with all agents *i* and *i_id_* as the search key. This hash table is used to generate the order in which agents act in the simulation.

*Female-Sensing*. At each cycle, all female agents in the swamp listen to the calls from all males and, according to their mating strategy, target one male to pursue.

Three female mating strategies were investigated. The first is the *best-of-n* strategy and requires females to target the male with the highest *pulsenumber* among the *n* closest males. The second strategy is the *minimum-threshold* strategy and it requires the female to target the closest male caller with *pulsenumber* greater than a minimum threshold θ. The third strategy is *random* choice of any male in the swamp. Male treefrogs call virtually continuously [24], so we assume that female agents re-assess male agents at each 1-sec update interval in the model.

We can define the first two strategies formally as in Scheutz et al [32]. Let

$c\left( f,X \right)=\left\{ m\in X \right|\neg\exists k\in X[d\left( k,f \right)<d\left( j,f \right)]\}$, i.e., $c(f,X)$ is a subset of *X* which contains the closest agents to *f*. Even though *c* frequently contains just one agent, if more than one agent has the same distance to *f* , then *c* contains multiple elements. Hence, $c^{n}(f,X)$ can be defined inductively as follows: $c^{0}\left( f,X \right)=\emptyset and c^{n+1}\left( f,X \right)=c^{n}\left( f,X \right)\cup\left\{ j\in X-c^{n}\left( f,X \right) \right|\neg\exists l\neq j (l\in\left( X-c^{n}\left( f,X \right) \right)\wedge d\left( l,f \right)< d\left( j,f \right))\}$.

- *best-of-n*. The selected male agent is $argmax_{m\in c^{n}\left( f,MALE \right)}\left( m_{pulsenumber} \right)$ i.e., the male with highest pulse number in the set of the closest n males.
- *min-threshold*. The selected male agent is $argmax_{m\in c(f,\{l\in MALE|l_{pulsenumber}\geq f_{\theta}}\left( m_{pulsenumber} \right),$where $f_{\theta}$ is the minimum threshold of female agent *f* , i.e., the closest male with a pulse number above the minimum threshold.

*Female-Acting.* After deciding which male to pursue, the female moves toward the targeted male in a straight line direct approach. On the same cycle, the female agent *i* can rotate, i.e., change her direction (*i_α_*) and move on that orientation according to her velocity (*i_v_*).

Female treefrogs showing a robust phonotaxis response to a male call in the field will move in short (1-2 sec) “walking bouts” immediately after the call and then pause and wait for the next call [33]. The distance moved during the walking bout varies, depending on the strength of the phonotaxis response. We computed an average rate of phonotaxis for female *H. versicolor* using data in [33]. This average value of 1.86cm/s incorporates the total time of both the walking bouts and pauses. Thus, for simplicity, we did not explicitly model the walk-pause-walk movement but instead assumed a constant rate of movement for females showing positive phonotaxis. In rare cases when females find no males above their *min-threshold*, they perform a random walk of 1.44 cm/sec for a single cycle [33].

*Male-Acting.* During this process, males verify if any female approached to a Euclidean distance less than or equal to *d_mate_*. Mating in grey treefrogs occurs when male and female first physically touch each other, so the mating distance was set to be within one body length (4 cm; [24]). Mating often lasts 4 or 5 hours so both sexes can effectively mate only once per night [18, 24]. Thus, agents that mate are removed from the simulation (removed from *femaleList*, *maleList* and *agentList*) and statistical information is stored.

*Check-Termination-Conditions.* Following the processes of *female-sensing*, *female-acting*, and *male-acting*, the model checks termination conditions. There are three ways to terminate the simulation: (1) all females have mated (i.e., *femaleList* is empty), (2) the maximum simulation time was reached (3600 cycles), or (3) there are no males with pulse number above the minimum threshold on the *min-threshold* strategy. When females are using the *min-threshold* strategy, if all remaining males in the swamp have a lower pulse number than the parameter θ, the females cannot mate and the simulation is finished. Therefore, for some simulations where the parameter θ is greater than the *pulsenumber* of all males, the simulation runs for just 1 cycle.

Distinct from most prior mate choice models, we focus on the decision-making process of females over a single night’s breeding activity and this governed our choice of the maximum simulation time. Female gray treefrogs are usually only present at the breeding site on the night they mate and oviposit. In the majority of cases, females mate on the first night they arrive [18, 22, 25]. It is thus reasonable to assume that female mate choice decisions are occurring over the time span of one night’s chorus, which lasts about 4 hours [24]. Decisions made on a single night might also have significant impact on male fitness. Many males will call for only one night in a season [24]. In one study of *H. chrysoscelis*, of 191 males marked during the season, 102 of those called for one night only [31]. Although we used a 4 hour simulation maximum in preliminary experiments, no simulation ever lasted more than 1 hour and thus 3600 sec was used here. Because all females in the simulation begin simultaneously, this is still a realistic time frame for mate choice behavior. The longer time for a natural chorus may be attributed to males and females entering the chorus at a variety of times, rather than synchronously.

*Set-Post-Simulation-State-Variables.* After the end of the simulation, the main controller executes this process to calculate all dependent variables from this run. These variables are stored as instance variables so they can be printed in the *Print-Results* submodel.

*Print-Results.* This is the last process called. It prints the independent parameters of the simulation followed by the dependent variables previously stored in the *Set-Post-Simulation-State-Variables* submodel.

**A.3 Model verification**

Initial verification included debugging, verifying implementation of conceptual models and calculations, and checking construct validity [34]. In addition, we investigated model implementation in a controlled verification environment. Each cycle in our model is divided into a period where female agents *sense* the environment and a second period where all agents *act*. Thus, we implemented an environment that receives *m* males and just one female with the initial position of the agents, mating strategy used by the female, call qualities of the males and the number of cycles executed by the simulation. As we used only one female, there is no female-female competition and thus we can test if the female is targeting the right male according to the strategy (i.e., the sense process was correctly implemented) and if the number of cycles needed to execute the simulation is also correct (i.e., the movement - or acting process - of the female was correctly implemented).

We defined 5 males with pulse numbers equal to {6, 18, 12, 24, 1} and locations equal to (333.33, 833.33), (666.66, 833.33), (333.33, 1666.66), (666.66, 1666.66), (500.00, 1250.00)}, respectively. We placed the female at a position in the set *S* ∈ {(333.33, 0.00), (666.66, 0.00), (1000.00, 833.33), (1000.00, 1666.66), (666.66, 2500.00), (333.33, 2500.00), (0.00, 1666.66), (0.00, 833.33)}.

After running the simulations, we verified the correctness of the sense process, because in all cases the female targeted the intended male to pursue. However, the acting process can only be verified for the *min-thresh* strategy, because in the *best-of-n* strategy, a female can “change her mind” if she approaches a better quality male while pursing another male. This does not occur in the *min-thresh* strategy because the female only targets another male if the previous male has already mated.

**Table A. Summary of the effects of female agent mate choice strategies across 3 different conditions for female updating of male agent locations (means ± 95% confidence intervals).**

| Strategy + Update Rule | Pulse Number of Male Mates | Distance Traveled by Females (cm) | Number of Males Targeted before Mating | Percent of Females that Mate |
| --- | --- | --- | --- | --- |
| Only *Best-of-n* updates at each time step^1^ |  |  |  |  |
| *Minimum-threshold* | 18.5 ± 0.1 | 287.6 ± 3.7 | 1.1 ± 0.01 | 61 |
| *Best-of-n* | 15.9 ± 0.1 | 588.1 ± 3.2 | 2.3 ± 0.01 | 100 |
| *Random* | 15.0 ± 0.2 | 1123.6 ± 5.8 | 1.3 ± 0.01 | 100 |
| No strategy updates at each time step^2^ |  |  |  |  |
| *Minimum-threshold* | 18.5 ± 0.1 | 287.6 ± 3.7 | 1.1 ± 0.01 | 61 |
| *Best-of-n* | 15.8 ± 0.1 | 533.5 ± 2.7 | 1.9 ± 0.01 | 100 |
| *Random* | 15.0 ± 0.2 | 1123.6 ± 10.2 | 1.3 ± 0.01 | 100 |
| No updating^3^ |  |  |  |  |
| *Minimum-threshold* | 18.4 ± 0.1 | 332.4 ± 4.7 | 1.1 ± 0.01 | 61 |
| *Best-of-n* | 15.8 ± 0.1 | 619.7 ± 3.7 | 1.9 ± 0.01 | 100 |
| *Random* | 15.0 ± 0.2 | 1280.2 ± 12.7 | 1.3 ± 0.01 | 100 |

^1^Female agents using *best-of-n* update at each time step and can “change their minds,” while those using *min-threshold* and *random* only update if target male is removed by mating.

^2^No female agents update at each time step but all update if target male is removed by mating, no matter where they are in the environment.

^3^All female agents travel to the location of the original target male and then update and change target if he is not there.

**Table B: Analysis of the distance traveled by females using the *minimum-threshold* strategy, divided by whether or not they mate (means ± 95% confidence intervals).**

| **Parameter settings** | **Distance traveled by females that mate** | **Distance traveled by females that do not** |
| --- | --- | --- |
| All thresholds combined | 423.3 ± 2.6 | 289.0 ± 12.8 |
| Threshold θ = 6 | 406.0 ± 4.2 | 1446.0 ± 51.0 |
| Threshold θ = 12 | 415.0 ± 5.0 | 402.0 ± 33.3 |
| Threshold θ = 18 | 434.3 ± 6.3 | 231.7 ± 20.5 |
| Threshold θ = 24 | 489.5 ± 9.4 | 163.0 ± 14.8 |

**Table C: Descriptions of model parameters for the treefrog mate choice simulation.**

| **Parameter** | **Description** | **Initial Value** | **Notes** |
| --- | --- | --- | --- |
| Female agents | | | |
| *size* | Given by *f_size_* | 5.28 cm | Constant; [31] |
| (*x,y*) | Position | Based on *f_δ_* | Calculated |
| *v* | Velocity given by *f_v_* | 1.86 cm/s during mate approach; 1.44 cm/s during random walks | Constant; [33] |
| α | Heading ∈ [0,360] degrees | 0 | Calculated |
| π(p) | Mating strategy π (*min-thresh* or *best-of-n*) with strategy parameter p | Given by *f_π_* together with strategy parameter values | Model-specific |
| *θ* | Minimum threshold value setting for the *min-thresh* strategy | 6 to 24 pulses per call in increments of 6 | Model-specific |
| *n* | Number of males sampled in the *best-of-n* strategy | 1 to 5 in increments of 1 | Model-specific |
| Male agents | | | |
| *size* | Given by *m_size_* | 4.72 cm | Constant; [24] |
| (*x,y*) | Position | Based on *m_δ_* | Constant; |
| *pulsenumber* | Number of pulses per call | Based on *µ_pulsenumber_* and *σ_pulsenumber_* | Constant; |
| Environment | | | |
| *E_x_* | Swamp width | 10m | Constant; [23] |
| *E_y_* | Swamp length | 25m | Constant; [23] |
| *d_mate_* | Mating distance | 4cm | Constant; [24] |
| *r_τ_* | Male territory radius | 50cm | Constant; [24] |
| *m* | Number of males | 25 | Constant; [26] |
| *f* | Number of females | 5 to 20 females varied in increments of 5 | Model-specific |
| *f_δ_* | Female distribution | Random at swamp edge | Constant |
| *m_δ_* | Male distribution | Random, Gaussian, or inverse Gaussian across the swamp | Model-specific |
| *µ_pulsenumber_* | Mean male pulse number | 6 to 24 pulses per call varied in increments of 6; *σ_pulsenumber_* = 2 | Model-specific |

Modified from [35].

**A.4 Supporting Information References**

1. Grimm V, Berger U, Bastiansen F, Eliassen S, Ginot V, Giske J, et al. A standard protocol for describing individual-based and agent-based models. Ecol Model. 2006;198(1-2):115-26. doi: 10.1016/j.ecolmodel.2006.04.023. PubMed PMID: WOS:000240823100008.

2. Grimm V, Berger U, DeAngelis DL, Polhill JG, Giske J, Railsback SF. The ODD protocol A review and first update. Ecol Model. 2010;221(23):2760-8. doi: 10.1016/j.ecolmodel.2010.08.019. PubMed PMID: WOS:000283980500004.

3. Scheutz M, Harris J. An overview of the SimWorld agent-based grid experimentation system. In: Werner DF, Kurowski K, Schott B, editors. Large-Scale Computing Techniques for Complex System Simulations: Wiley; 2011.

4. Gibson RM, Langen TA. How do animals choose their mates? Trends Ecol Evol. 1996;11(11):468-70. PubMed PMID: ISI:A1996VN15900009.

5. Schwartz JJ, Buchanan BW, Gerhardt HC. Female mate choice in the gray treefrog (*Hyla versicolor*) in three experimental environments. Behav Ecol Sociobiol. 2001;49(6):443-55. PubMed PMID: ISI:000168778400002.

6. Schwartz JJ, Huth K, Hutchin T. How long do females really listen? Assessment time for female mate choice in the grey treefrog, *Hyla versicolor*. Anim Behav. 2004;68:533-40. PubMed PMID: ISI:000224060100011.

7. Bush SL, Gerhardt HC, Schul J. Pattern recognition and call preferences in treefrogs (Anura : Hylidae): a quantitative analysis using a no-choice paradigm. Anim Behav. 2002;63:7-14. PubMed PMID: ISI:000173746700002.

8. Gerhardt HC, Tanner SD, Corrigan CM, Walton HC. Female preference functions based on call duration in the gray tree frog (*Hyla versicolor*). Behav Ecol. 2000;11(6):663-9. PubMed PMID: ISI:000165878000012.

9. Jennions MD, Petrie M. Variation in mate choice and mating preferences: A review of causes and consequences. Biol Rev Cambridge Philosophic Soc. 1997;72(2):283-327. PubMed PMID: ISI:A1997WY44300003.

10. Ryan MJ, Keddyhector A. Directional patterns of female mate choice and the role of sensory biases. Am Nat. 1992;139:S4-S35. PubMed PMID: ISI:A1992HM36800002.

11. Gerhardt HC. The evolution of vocalization in frogs and toads. Annu Rev Ecol Evol Syst. 1994;25:293-324. PubMed PMID: ISI:A1994PU88300012.

12. Welch AM, Semlitsch RD, Gerhardt HC. Call duration as an indicator of genetic quality in male gray tree frogs. Science. 1998;280(5371):1928-30. PubMed PMID: ISI:000074323800061.

13. Gerhardt HC. Phonotactic selectivity in two cryptic species of gray treefrogs: effects of differences in pulse rate, carrier frequency and playback level. J Exp Biol. 2008;211(16):2609-16. PubMed PMID: ISI:000258297000011.

14. Witte K, Ryan MJ, Wilczynski W. Changes in the frequency structure of a mating call decrease its attractiveness to females in the cricket frog *Acris crepitans blanchardi*. Ethology. 2001;107(8):685-99. PubMed PMID: ISI:000171351400003.

15. Parris KM. More bang for your buck: the effect of caller position, habitat and chorus noise on the efficiency of calling in the spring peeper. Ecol Model. 2002;156(2-3):213-24. PubMed PMID: ISI:000178386700009.

16. Greenfield MD, Rand AS. Frogs have rules: Selective attention algorithms regulate chorusing in *Physalaemus pustulosus* (Leptodactylidae). Ethology. 2000;106(4):331-47. PubMed PMID: ISI:000086783400004.

17. Gerhardt HC, Klump GM. Masking of acoustic signals by the chorus background noise in the green tree frog - a limitation on mate choice. Anim Behav. 1988;36:1247-9. PubMed PMID: ISI:A1988P531400037.

18. Morris MR. Female choice of large males in the treefrog *Hyla chrysoscelis* - the importance of identifying the scale of choice. Behav Ecol Sociobiol. 1989;25(4):275-81. PubMed PMID: ISI:A1989AR53300006.

19. Grafe TU. Costs and benefits of mate choice in the lek-breeding reed frog, *Hyperolius marmoratus*. Anim Behav. 1997;53:1103-17. PubMed PMID: ISI:A1997XA36700023.

20. Murphy CG, Gerhardt HC. Mate sampling by female barking treefrogs (*Hyla gratiosa*). Behav Ecol. 2002;13(4):472-80. doi: 10.1093/beheco/13.4.472. PubMed PMID: WOS:000176665200007.

21. Welch AM. Genetic benefits of a female mating preference in gray tree frogs are context-dependent. Evolution. 2003;57(4):883-93. PubMed PMID: ISI:000182815100017.

22. Sullivan BK, Hinshaw SH. Female choice and selection on male calling behavior in the gray treefrog *Hyla versicolor*. Anim Behav. 1992;44(4):733-44. PubMed PMID: ISI:A1992JY67800011.

23. Fellers GM. Mate selection in the gray treefrog, *Hyla versicolor*. Copeia. 1979;(2):286-90. PubMed PMID: ISI:A1979GW13300014.

24. Fellers GM. Aggression, territoriality, and mating behavior in North American treefrogs. Anim Behav. 1979;27(FEB):107-19. PubMed PMID: ISI:A1979GS52600006.

25. Godwin GJ, Roble SM. Mating success in male treefrogs, *Hyla chrysoscelis* (Anura, Hylidae). Herpetologica. 1983;39(2):141-6. PubMed PMID: ISI:A1983RA55400008.

26. Ptacek MB. Calling sites used by male gray treefrogs, *Hyla versicolor* and *Hyla chrysoscelis*, in sympatry and allopatry in Missouri. Herpetologica. 1992;48(4):373-82. PubMed PMID: ISI:A1992KB08400001.

27. Bertram S, Berrill M, Nol E. Male mating success and variation in chorus attendance within and among breeding seasons in the gray treefrog (*Hyla versicolor*). Copeia. 1996;(3):729-34. PubMed PMID: ISI:A1996VB03300026.

28. Hausfater G, Gerhardt HC, Klump GM. Parasites and mate choice in gray treefrogs, *Hyla versicolor*. Am Zool. 1990;30(2):299-311. PubMed PMID: ISI:A1990DV88100009.

29. Gerhardt HC, Daniel, R.E., Perril, S.A., and Schramm, S. Mating behavior and male mating success in the green treefrog. Anim Behav. 1987;35(5):1490-503.

30. Gatz AJ. Size selective mating in *Hyla versicolor* and *Hyla crucifer*. J Herpetol. 1981;15(1):114-6. PubMed PMID: ISI:A1981LF56200016.

31. Ritke ME, Semlitsch RD. Mating behavior and determinants of male mating success in the gray treefrog, *Hyla chrysoscelis*. Can J Zool. 1991;69(1):246-50. PubMed PMID: ISI:A1991EX56500037.

32. Scheutz M, Smiley M, Boyd SK. Exploring male spatial placement strategies in a biologically plausible mating task. 2013 IEEE Symposium on Artificial Life. IEEE Symposium on Artificial Life. New York: IEEE; 2013. p. 113-9.

33. Beckers OM, Schul J. Phonotaxis in *Hyla versicolor* (Anura, Hylidae): the effect of absolute call amplitude. J Comp Physiol A -Neuroethol Sens Neural Behav Physiol. 2004;190(11):869-76. PubMed PMID: ISI:000225237900002.

34. Kennedy RC, Xiang X, Cosimano TF, Madey GR, Cabaniss SE, editors. Verification and validation of agent-based and equation-based simulations: A comparison. Agent-Directed Simulation; 2006; Huntsville, AL2006.

35. Scheutz M, Harris J, Boyd SK. How to pick the right one: Investigating tradeoffs among female mate choice strategies in treefrogs. In: Doncieux S, Girard B, Guillot A, Hallam J, Meyer JA, Mouret JB, editors. From Animals to Animats 11. Lecture Notes in Artificial Intelligence. 62262010. p. 618-27.

**A.5 Model Code**

import java.util.*;

/**

* Controls the initial configuration of the territories in the swamp, places

* the males according to the territories, places the females at the edges of

* the swamp, updates the frogs' situation on every simulation

*/

class SwampNR {

public static void main(String [] args) {

Scanner in = new Scanner(System.in);

SwampNR simulation = new SwampNR();

long seed;

System.out.println("Seed: ");

seed = in.nextLong();

int femaleStrategy;

System.out.println("Female Strategy - 0 (best of n) 1 (minimum threshold) 2 (random): ");

femaleStrategy = in.nextInt();

int strategyParam;

System.out.println("Strategy Parameter: ");

strategyParam = in.nextInt();

int numFemales;

System.out.println("Number of Females: ");

numFemales = in.nextInt();

int meanPulseNumber;

System.out.println("Mean Pulse Number: ");

meanPulseNumber = in.nextInt();

int maleDistribution;

System.out.println("MaleDistribution - 0 (gaussian) 1 (inversegaussian) 2 (grid) 4 (random): ");

maleDistribution = in.nextInt();

simulation.run(seed, maleDistribution, meanPulseNumber, numFemales, femaleStrategy, strategyParam);

}

/*************************************

********** PRIVATE CLASSES **********

*************************************/

/**

* Class that encapsulates a mating

*/

private static class MateEvent {

int cycle;

String femaleID;

String maleID;

int pulseNumber;

public MateEvent(int cycle, String femaleID, String maleID, int pulseNumber) {

this.cycle = cycle;

this.femaleID = femaleID;

this.maleID = maleID;

this.pulseNumber = pulseNumber;

}

}

/*************************************

*************** ENUMS ***************

*************************************/

//Male distributions

public enum MaleDistributions {

gaussian(0),

inversegaussian(1),

randomm(4);

private int numVal;

private MaleDistributions(int numVal) {

this.numVal = numVal;

}

public int getNumVal() {

return numVal;

}

}

//Female strategies

public enum MatingRules {

bestOfN(0),

minThreshold(1),

random(2);

private int numVal;

private MatingRules(int numVal) {

this.numVal = numVal;

}

public int getNumVal() {

return numVal;

}

}

//Female Distribution

public enum FemaleDistributions {

random(0);

private int numVal;

private FemaleDistributions(int numVal) {

this.numVal = numVal;

}

public int getNumVal() {

return numVal;

}

}

/*************************************

******* SIMULATION CONSTANTS ********

*************************************/

// sizes

public static final double MATINGRANGE = 4; //Distance to mate

public static final double TERRITORY = 50; //Minimum distance between two males

/*

* constants for female distributions

*/

private static final double MEANX = 0.5;

private static final double MEANY = 0.5;

// MS: these are used by the male frogs to generate their calls

public static int minPulseNumber = 1;

public static int maxPulseNumber = 40;

/*************************************

******* INDEPENDENT VARIABLES *******

*************************************/

public long seed = 1 + (new java.util.Date()).getTime() % 10000;

protected SpecialRandom random = null; //

// added a maximum cycle number

public int runCycles = 1200;

//Swamp size

public int SwampWidth = 1000;

public int SwampHeight = 2500;

// Quantity of males in the swamp

public int numMales = 25;

// Quantity of females in the swamp

public int numFemales = 10;

//Mating strategy

public MatingRules female_rule_mating = MatingRules.minThreshold; // (0=best of N,1=Min thresh ,2=random)

//Integer representing the mating strategy

public int rule_matingInt = -1;

//Strategy parameters

public int nBestN = 3; // only to be used with rule_mating =0

public int minThreshold = 16; // only to be used with female_rule_mating =1

//Variables to generate pulse number for males

public int meanPulseNumber = 16;

public int stddevPulseNumber = 2;

//Male distribution

public MaleDistributions maleDistribution = MaleDistributions.gaussian;

//Integer representing the male distribution

public int maleDistributionInt = -1;

//Female distribution

public FemaleDistributions femaleDistribution = FemaleDistributions.random;

//Integer representing the female distribution

public int femaleDistributionInt = -1;

/*************************************

******** DEPENDENT VARIABLES ********

*************************************/

//Time spent to mate on average

public double averageMateTime = -1;

//Quantity of mating males

public int numMated = 0;

//Average pulses per call of mated males

public double averageMatedPulseNumber = -1;

//Average of the distance travelled by females

public double averageFemalesDistanceTravelled = -1;

//Last simulation cycle

public static int cycle = 0;

//String with the males initial and final positions

public String maleListStr = "";

//String with the females initial and final positions

public String femaleListStr = "";

/*************************************

******** AUXILIARY VARIABLES ********

*************************************/

// bookkeeping lists

private MaleArrayList maleList = new MaleArrayList(new ArrayList<Male>());

private FemaleArrayList femaleList = new FemaleArrayList(new ArrayList<Female>());

private Hashtable<String, TreeFrog> agentList = new Hashtable<>();

private ArrayList<Male> terrList = new ArrayList<>();

// list that stores the mating events

private static ArrayList<MateEvent> reportMated = new ArrayList<>();

//Count of matings

public static int matedCount = 0;

/*************************************

********* AUXILIARY METHODS *********

*************************************/

public static void reportMating(String femaleID, String maleID, int pulseNumber) {

reportMated.add(new MateEvent(SwampNR.cycle, femaleID, maleID, pulseNumber));

}

/*************************************

*********** CLASS METHODS ***********

*************************************/

/**

* Creates a new Swamp environment

*/

public SwampNR() {

}

/**

* Run a simulation with defined parameters

* @param seed Seed to generate all random numbers

* @param maleDistribution Distribution of males in the swamp (0-gaussian, 1-inversegaussian, 4-random)

* @param meanPulseNumber Mean of the gaussian distribution of pulse numbers

* @param numberOfFemales Number of females in the swamp

* @param femaleStrategy Strategy played by all females in the swamp

* @param strategyParam Parameter related to the strategy

*/

public void run(long seed, int maleDistribution, int meanPulseNumber, int numberOfFemales, int femaleStrategy, int strategyParam) {

setParameters(seed, maleDistribution, meanPulseNumber, numberOfFemales, femaleStrategy, strategyParam);

createAgents();

boolean terminate = false;

SwampNR.cycle = 0;

while(!terminate) {

//Females sensing the swamp

for(Female female : femaleList.get()) {

female.sense(agentList);

}

//Agents acting

for(TreeFrog frog : agentList.values()) {

frog.act(agentList);

}

++SwampNR.cycle;

terminate = checkTerminationConditions();

}

setPostSimulationStateVariables();

printResults();

}

/**

* Set the parameter in the simulation

* @param seed Seed to generate all random numbers

* @param maleDistribution Distribution of males in the swamp (0-gaussian, 1-inversegaussian, 4-random)

* @param meanPulseNumber Mean of the gaussian distribution of pulse numbers

* @param numberOfFemales Number of females in the swamp

* @param femaleStrategy Strategy played by all females in the swamp

* @param strategyParam Parameter related to the strategy

*/

private void setParameters(long seed, int maleDistribution, int meanPulseNumber, int numberOfFemales, int femaleStrategy, int strategyParam) {

this.seed = seed;

this.random = new SpecialRandom(seed); //reset random number generator

this.maleDistributionInt = maleDistribution;

this.meanPulseNumber = meanPulseNumber;

this.numFemales = numberOfFemales;

this.rule_matingInt = femaleStrategy;

switch (rule_matingInt) {

case 0:

this.nBestN = strategyParam;

break;

case 1:

this.minThreshold = strategyParam;

break;

default:

}

}

/**

* Get the results from the matings and print the

* average of females switches and average pulse number

* of males.

* @param femaleListStr String containing the locations of females for every heading change

*/

private void getFemales(String femaleListStr)

{

Map<String, ArrayList<Map<String, Integer> > > l_females = new HashMap<>();

Map<String, Integer> l_males = new HashMap<>();

int l_pointer = 0;

StringBuilder l_currentFem = new StringBuilder();

boolean l_pipe = false;

while(l_pointer < femaleListStr.length())

{

boolean l_finishName = false;

if(femaleListStr.charAt(l_pointer) == 'f')

{

StringBuilder l_id = new StringBuilder();

while(femaleListStr.charAt(l_pointer) != '[')

{

l_id.append(femaleListStr.charAt(l_pointer));

++l_pointer;

}

l_currentFem = l_id;

l_finishName = true;

}

else if(femaleListStr.charAt(l_pointer) == '[')

{

++l_pointer;

if(femaleListStr.charAt(l_pointer) == '(')

{

++l_pointer;

StringBuilder l_number = new StringBuilder();

double l_firstNumber;

double l_secondNumber;

double l_thirdNumber;

double l_forthNumber;

while(femaleListStr.charAt(l_pointer) != '#')

{

l_number.append(femaleListStr.charAt(l_pointer));

++l_pointer;

}

l_firstNumber = Double.parseDouble(l_number.toString());

l_number = new StringBuilder();

++l_pointer;

while(femaleListStr.charAt(l_pointer) != ')')

{

l_number.append(femaleListStr.charAt(l_pointer));

++l_pointer;

}

l_secondNumber = Double.parseDouble(l_number.toString());

l_number = new StringBuilder();

l_pointer += 3;

while(femaleListStr.charAt(l_pointer) != '#')

{

l_number.append(femaleListStr.charAt(l_pointer));

++l_pointer;

}

l_thirdNumber = Double.parseDouble(l_number.toString());

l_number = new StringBuilder();

++l_pointer;

while(femaleListStr.charAt(l_pointer) != ')')

{

l_number.append(femaleListStr.charAt(l_pointer));

++l_pointer;

}

l_forthNumber = Double.parseDouble(l_number.toString());

}

}

else if(femaleListStr.charAt(l_pointer) == '{' || l_pipe == true)

{

if(!l_pipe)

{

++l_pointer;

}

l_pipe = false;

if(femaleListStr.charAt(l_pointer) == 'm')

{

StringBuilder l_maleName = new StringBuilder();

while(femaleListStr.charAt(l_pointer) != '*')

{

l_maleName.append(femaleListStr.charAt(l_pointer));

++l_pointer;

}

++l_pointer;

StringBuilder l_number = new StringBuilder();

Integer l_ppc;

while(femaleListStr.charAt(l_pointer) != '}')

{

if(femaleListStr.charAt(l_pointer) != '|')

{

l_number.append(femaleListStr.charAt(l_pointer));

++l_pointer;

}

else

{

l_pipe = true;

break;

}

}

l_ppc = Integer.parseInt(l_number.toString());

if(l_females.containsKey(l_currentFem.toString()))

{

Map<String, Integer> map = new HashMap<>();

map.put(l_maleName.toString(), l_ppc);

ArrayList<Map<String, Integer> > l_array = l_females.get(l_currentFem.toString());

l_array.add(map);

}

else

{

Map<String, Integer> map = new HashMap<>();

map.put(l_maleName.toString(), l_ppc);

ArrayList<Map<String, Integer> > l_array = new ArrayList<>();

l_array.add(map);

l_females.put(l_currentFem.toString(), l_array);

}

l_males.put(l_maleName.toString(), l_ppc);

if(!l_pipe)

{

l_currentFem = new StringBuilder();

}

}

}

if(!l_finishName)

{

++l_pointer;

}

}

int switches = 0;

for (Map.Entry<String, ArrayList<Map<String, Integer> > > entry : l_females.entrySet())

{

switches += entry.getValue().size();

}

int sumPN = 0;

for (Map.Entry<String, Integer> entry : l_males.entrySet())

{

sumPN += entry.getValue();

}

System.out.print((double)switches/numFemales + ",");

System.out.print((double)sumPN/l_males.size() + ",");

}

/**

* Print results of the simulation

*/

public void printResults() {

System.out.println("Replicate,Strategy,Parameter,NumFemales,MalePulses,MaleDistribution,NumMated,AvgMatedPulsesPerCall,AvgMateTime,AvgDistanceTravelled,AvgFemalesSwitches,AvgPulseNumber,LastCycle");

System.out.print(seed + ",");

switch (rule_matingInt) {

case 0:

System.out.print("BestOfN,");

switch(nBestN) {

case 1:

System.out.print("BestOf1,");

break;

case 2:

System.out.print("BestOf2,");

break;

case 3:

System.out.print("BestOf3,");

break;

case 4:

System.out.print("BestOf4,");

break;

case 5:

System.out.print("BestOf5,");

break;

}

break;

case 1:

System.out.print("MinThresh,");

System.out.print("MinThresh " + minThreshold + ",");

break;

case 2:

System.out.print("Random,");

System.out.print("Random,");

break;

default:

}

System.out.print(numFemales + ",");

System.out.print(meanPulseNumber + ",");

switch (maleDistributionInt) {

case 0:

System.out.print("gaussian,");

break;

case 1:

System.out.print("inversegaussian,");

break;

case 2:

System.out.print("grid,");

break;

case 4:

System.out.print("random,");

break;

default:

}

System.out.print(numMated + ",");

if(averageMatedPulseNumber >= 0.0) {

System.out.print(averageMatedPulseNumber + ",");

}

else {

System.out.print("NaN,");

}

if(averageMateTime >= 0.0) {

System.out.print(averageMateTime + ",");

}

else {

System.out.print("NaN,");

}

System.out.print(averageFemalesDistanceTravelled + ",");

getFemales(femaleListStr);

System.out.println(cycle);

}

/**

* Verify if the current cycle is greater than

* runCycles. Of if all females mated.

* @return true if the simulation is over

*/

public boolean checkTerminationConditions() {

if(SwampNR.cycle > runCycles) {

return true;

}

boolean noMoreMales = true;

boolean allMated = true;

for (Female female : femaleList.get()) {

if(!female.getTreeFrogState().mated) {

allMated = false;

if(!female.getTreeFrogState().currentFrogToPursue.equals("none")) {

noMoreMales = false;

}

}

}

return allMated || noMoreMales;

}

/**

* Creates the frogs used on the simulation

*/

protected void createAgents() {

SwampDimensions.setSwampDimensions(SwampWidth, SwampHeight);

runCycles = 3600;

switch (rule_matingInt) {

case 0:

female_rule_mating = MatingRules.bestOfN;

break;

case 1:

female_rule_mating = MatingRules.minThreshold;

break;

case 2:

female_rule_mating = MatingRules.random;

break;

default:

}

switch (maleDistributionInt) {

case 0:

maleDistribution = MaleDistributions.gaussian;

break;

case 1:

maleDistribution = MaleDistributions.inversegaussian;

break;

case 4:

maleDistribution = MaleDistributions.randomm;

break;

default:

}

switch (femaleDistributionInt) {

case 0:

femaleDistribution = FemaleDistributions.random;

break;

}

initializeFrogs();

}

/**

* Initialize the frogs used in the simulation

*/

private void initializeFrogs() {

for (int i = 0; i < numMales; i++) {

maleList.get().add(new Male("male"+(i + 1), 0.0, 0.0, random.nextLong(), meanPulseNumber, stddevPulseNumber));

}

// initialize location only after all pulses have been assigned...

for (Male male : maleList.get()) {

initialize(male);

}

if (femaleDistribution == FemaleDistributions.random) {

initializeFemales();

for (Female tempFemale : femaleList.get()) {

initializeFemaleParameters(tempFemale);

}

} else {

System.err.println("SwampNR(initializeFrogs) UNKNOWN FEMALE DISTRIBUTION "

+ femaleDistribution);

System.exit(-1);

}

for (Male tempMale : maleList.get()) {

agentList.put(tempMale.getTreeFrogState().getAgentID(), tempMale);

}

for (Female tempFemale : femaleList.get()) {

agentList.put(tempFemale.getTreeFrogState().getAgentID(), tempFemale);

}

}

/**

* Set the results into the dependent variables after the end of the simulation

*/

public void setPostSimulationStateVariables() {

matedCount = 0;

averageMateTime = -1;

averageMatedPulseNumber = -1;

averageFemalesDistanceTravelled = -1;

int totalpn = 0;

for (Male m : maleList.get()) {

if (m.getTreeFrogState().mated == true) {

matedCount++;

totalpn += m.getPulseNumber();

}

}

if (matedCount > 0) {

int matedTotalTime = 0; //total time

for (MateEvent m : reportMated) {

matedTotalTime += m.cycle;

}

averageMateTime = (double) ((double) matedTotalTime / (double) matedCount);

averageMatedPulseNumber = (double) totalpn / (double) matedCount;

}

averageFemalesDistanceTravelled = 0;

int femaleListSize = 0;

for (Female f : femaleList.get()) {

averageFemalesDistanceTravelled += f.getDistanceTravelled();

++femaleListSize;

}

averageFemalesDistanceTravelled /= femaleListSize;

numMated = matedCount;

maleListStr = maleList.toString();

femaleListStr = femaleList.toString();

}

/**

* Checks if the frog being compared is within the range specified

* @param frog Current frog

* @param toCompare List of other frogs to compare

* @param range Distance to check

* @return true if the frog is within the specified range

*/

private boolean oneWithinRange(TreeFrog frog, ArrayList toCompare,

double range) {

int listSize = toCompare.size();

for (int f = 0; f < listSize; f++) {

TreeFrog frogToCompare = (TreeFrog) toCompare.get(f);

double dist = TreeFrog.withinDistance(frog, frogToCompare);

if (dist <= range) {

return true;

}

}

return false;

}

/**********************************************************

**************** MEMORY INITIALIZATION *******************

**********************************************************/

/**

* Initialize males according to the male distribution

* @param thisMale Male to being initialized

*/

private void initialize(Male thisMale) {

boolean outside = true;

while (outside) {

if(maleDistribution == MaleDistributions.gaussian)

{

initializeGaussian(thisMale);

}

else if(maleDistribution == MaleDistributions.inversegaussian)

{

initializeBimodal(thisMale);

}

else if(maleDistribution == MaleDistributions.randomm)

{

initializeRandom(thisMale);

}

//Verify bounds

outside = false;

if (thisMale.getX() > SwampDimensions.getSwampWidth()) {

outside = true;

}

if (thisMale.getY() > SwampDimensions.getSwampHeight()) {

outside = true;

}

}

}

/**

* Initialize male at random distribution

* @param thisMale Male to being initialized

*/

private void initializeRandom(Male thisMale) {

boolean overlap = true;

double dist = 2 * TERRITORY;

while (overlap) {

// draw random sample

double tempCoord = Math.abs(thisMale.random.nextDouble() * SwampDimensions.getSwampWidth());

while (tempCoord - TERRITORY < 0 || tempCoord + TERRITORY > SwampDimensions.getSwampWidth()) {

tempCoord = Math.abs(thisMale.random.nextDouble() * SwampDimensions.getSwampWidth());

}

thisMale.setX(tempCoord);

tempCoord = Math.abs(thisMale.random.nextDouble() * SwampDimensions.getSwampHeight());

while (tempCoord - TERRITORY < 0 || tempCoord + TERRITORY > SwampDimensions.getSwampHeight()) {

tempCoord = Math.abs(thisMale.random.nextDouble() * SwampDimensions.getSwampHeight());

}

thisMale.setY(tempCoord);

overlap = oneWithinRange(thisMale, terrList, dist);

}

terrList.add(thisMale);

}

/**

* Places the males according to the existing males in the swamp using

* Gaussian distribution

* @param thisMale Male to being initialized

*/

private void initializeGaussian(Male thisMale)// obj:nest_site,parents)

{

boolean overlap = true;

double dist = 2 * TERRITORY;

int overlapCount = 0;

while (overlap) {

overlapCount++;

// draw Gaussian sample

double tempCoord = Math.abs(thisMale.random.nextGaussian(SwampDimensions.getSwampWidth()

* MEANX, SwampDimensions.getSwampWidth() * MEANX / 2));

while (tempCoord - TERRITORY < 0 || tempCoord + TERRITORY > SwampDimensions.getSwampWidth()) {

tempCoord = Math.abs(thisMale.random.nextGaussian(SwampDimensions.getSwampWidth() * MEANX,

SwampDimensions.getSwampWidth() * MEANX / 2));

}

thisMale.setX(tempCoord);

tempCoord = Math.abs(thisMale.random.nextGaussian(SwampDimensions.getSwampHeight() * MEANY,

SwampDimensions.getSwampHeight() * MEANY / 2));

while (tempCoord - TERRITORY < 0 || tempCoord + TERRITORY > SwampDimensions.getSwampHeight()) {

tempCoord = Math.abs(thisMale.random.nextGaussian(SwampDimensions.getSwampHeight() * MEANX,

SwampDimensions.getSwampHeight() * MEANX / 2));

}

thisMale.setY(tempCoord);

overlap = oneWithinRange(thisMale, terrList, dist);

if (overlapCount > 50) {

System.err.println("50 overlaps...somethng wasn't reset.");

}

}

terrList.add(thisMale);

}

/**

* Places the males according to the existing males in the swamp using 2

* Gaussian distributions to better simulate the bimodal distribution

*

* g(0,STD) and another one g(maxX or maxY,STD)

*

* MALES must be within the swamp limits, including their territory

* @param thisMale Male to being initialized

*/

private void initializeBimodal(Male thisMale) {

boolean overlap = true;

double dist = 2 * TERRITORY;

while (overlap) {

double tempCoord;

if (thisMale.random.nextDouble() < 0.5) {

tempCoord = Math.abs(thisMale.random.nextGaussian(SwampDimensions.getSwampWidth(), SwampDimensions.getSwampWidth()

* MEANX / 2));

while (tempCoord - TERRITORY < 0

|| tempCoord + TERRITORY > SwampDimensions.getSwampWidth()) {

tempCoord = Math.abs(thisMale.random.nextGaussian(SwampDimensions.getSwampWidth(),

SwampDimensions.getSwampWidth() * MEANX / 2));

}

} else {

tempCoord = Math.abs(thisMale.random.nextGaussian(0, SwampDimensions.getSwampWidth()

* MEANX / 2));

while (tempCoord - TERRITORY < 0

|| tempCoord + TERRITORY > SwampDimensions.getSwampWidth()) {

tempCoord = Math.abs(thisMale.random.nextGaussian(0, SwampDimensions.getSwampWidth()

* MEANX / 2));

}

}

thisMale.setX(tempCoord);

if (thisMale.random.nextDouble() < 0.5) {

tempCoord = Math.abs(thisMale.random.nextGaussian(SwampDimensions.getSwampHeight(), SwampDimensions.getSwampHeight()

* MEANY / 2));

while (tempCoord - TERRITORY < 0

|| tempCoord + TERRITORY > SwampDimensions.getSwampHeight()) {

tempCoord = Math.abs(thisMale.random.nextGaussian(SwampDimensions.getSwampHeight(),

SwampDimensions.getSwampHeight() * MEANX / 2));

}

} else {

tempCoord = Math.abs(thisMale.random.nextGaussian(0, SwampDimensions.getSwampHeight()

* MEANY / 2));

while (tempCoord - TERRITORY < 0

|| tempCoord + TERRITORY > SwampDimensions.getSwampHeight()) {

tempCoord = Math.abs(thisMale.random.nextGaussian(0, SwampDimensions.getSwampHeight()

* MEANX / 2));

}

}

thisMale.setY(tempCoord);

overlap = oneWithinRange(thisMale, terrList, dist);

}

terrList.add(thisMale);

}

/**

* Place the females using formula: if random <

* SwampHeight/(SwampHeight-SwampWidth) place it with 50% chance East or

* West otherwise, with 50% chance North or South

*/

private void initializeFemales() {

char place;

double whereRandom = (double) SwampDimensions.getSwampHeight() / (double) (SwampDimensions.getSwampHeight() + SwampDimensions.getSwampWidth());

Female thisFemale = null;

double tempCoord;

for (int fno = 1; fno < numFemales + 1;) {

double rand = random.nextDouble();

if (rand < (1 - whereRandom)) // E-W

{

if (random.nextDouble() < 0.5) {

place = 'n';

} else {

place = 's';

}

} else // N-S

// if (rand < whereRandom) {

if (random.nextDouble() < 0.5) {

place = 'e';

} else {

place = 'w';

}

// }

switch(place) {

case 'n':

thisFemale = new Female("female"+(fno++), 0.0, 0.0, random.nextLong());

tempCoord = Math.abs(thisFemale.random.nextDouble() * SwampDimensions.getSwampWidth());

while (tempCoord < 0 || tempCoord > SwampDimensions.getSwampWidth()) {

tempCoord = Math.abs(thisFemale.random.nextDouble() * SwampDimensions.getSwampWidth());

}

if (tempCoord < 1.0) {

tempCoord *= SwampDimensions.getSwampWidth();

}

thisFemale.setX(tempCoord);

thisFemale.setY(SwampDimensions.getSwampHeight() - 0.1);

femaleList.get().add(thisFemale);

break; // north

case 's':

thisFemale = new Female("female"+(fno++), 0.0, 0.0, random.nextLong());

tempCoord = Math.abs(thisFemale.random.nextDouble() * SwampDimensions.getSwampWidth());

while (tempCoord < 0 || tempCoord > SwampDimensions.getSwampWidth()) {

tempCoord = Math.abs(thisFemale.random.nextDouble() * SwampDimensions.getSwampWidth());

}

if (tempCoord < 1.0) {

tempCoord *= SwampDimensions.getSwampWidth();

}

thisFemale.setX(tempCoord);

thisFemale.setY(0.0);

femaleList.get().add(thisFemale);

break;// south

case 'e':

thisFemale = new Female("female"+(fno++), 0.0, 0.0, random.nextLong());

tempCoord = Math.abs(thisFemale.random.nextDouble() * SwampDimensions.getSwampHeight());

while (tempCoord < 0 || tempCoord > SwampDimensions.getSwampHeight()) {

tempCoord = Math.abs(thisFemale.random.nextDouble() * SwampDimensions.getSwampHeight());

}

if (tempCoord < 1.0) {

tempCoord *= SwampDimensions.getSwampHeight();

}

thisFemale.setY(tempCoord);

thisFemale.setX(SwampDimensions.getSwampWidth() - 0.1);

femaleList.get().add(thisFemale);

break; // East

case 'w':

thisFemale = new Female("female"+(fno++), 0.0, 0.0, random.nextLong());

tempCoord = Math.abs(thisFemale.random.nextDouble() * SwampDimensions.getSwampHeight());

while (tempCoord < 0 || tempCoord > SwampDimensions.getSwampHeight()) {

tempCoord = Math.abs(thisFemale.random.nextDouble() * SwampDimensions.getSwampHeight());

}

if (tempCoord < 1.0) {

tempCoord *= SwampDimensions.getSwampHeight();

}

thisFemale.setY(tempCoord);

thisFemale.setX(0.0);

femaleList.get().add(thisFemale);

break;// West

default:

}

if(thisFemale != null) {

thisFemale.setInitialX(thisFemale.getX());

thisFemale.setInitialY(thisFemale.getY());

}

}// for

}// initializeFemales

/**

* This method initialize the mating parameters of the females.

* @param female Female that need to be initialized

*/

private void initializeFemaleParameters(Female female) {

female.setMatingRule(female_rule_mating);

female.setNtoMate(nBestN);

female.setMinThreshold(minThreshold);

}

}

/**

* Wrapper for a female frog

*/

class Female extends TreeFrog {

/**

* Class that stores a plan to approach a potential male

*/

private class Plan {

boolean attemptMate = false;

TreeFrogState potentialMate = null;

}

//Speed when approaching a male

private static double speedPhonotaxis = 1.86;// cm/sec

private static double speedRandom = 1.44;// cm/sec

private int randomNegX = 0;

private int randomNegY = 0;

//Heading angle

private double headingAngle = 0.0;

//Strategy

private SwampNR.MatingRules matingRule;

//Parameter n of best-of-n strategy

private int NTOMATE = 5;

//Parameter theta of min-thresh strategy

private int minThreshold;

//Distance traveled during the simulation

private double distanceTravelled = 0.0;

//Male selected before the current male

private TreeFrogState chosenBefore = null;

//Aux variables to calculate the heading

private double headingX = -1;

private double headingY = -1;

//Aux variable to update subseq variables correctly

//in the first time updateDistance() is called

private boolean updatedSubSeq = false;

//Last position of the female before changing heading

private double subseqX = 0, subseqY = 0;

//Initial position of female

private double initialX = 0, initialY = 0;

//Plan containing the male to pursue

private Plan myPlan = new Plan();

//List of chosen males

private ArrayList<String> listOfChosenMales = new ArrayList<>();

/**

* Creates the frog in the given position Max: changed id to a String

* @param agentID ID of the frog

* @param iX X Position of the frog

* @param iY Y Position of the frog

* @param seed Random seed

*/

public Female(String agentID, double iX, double iY, long seed) {

super(agentID,

iX,

iY,

seed);

updatedSubSeq = false;

size = 5.28; // default female size, cms

}

/**

* Female senses the world and chooses a male to pursue

* @param maleList all males that the female can interact with physically

*/

public void sense(Hashtable<String, TreeFrog> maleList) {

if ((!isAlive()) || this.getTreeFrogState().mated) {

return;

}

// See if mating occurred last step

if (myPlan.potentialMate != null) {

if (myPlan.potentialMate.lastMateID.equals(this.getTreeFrogState().getAgentID())) {

this.getTreeFrogState().mated = true;

updateDistance();

myPlan.potentialMate = null;

return;

}

}

// determine if the female should mate

ArrayList<TreeFrogState> livingMales = new ArrayList<>();

for (TreeFrog potentialMate : maleList.values()) {

if(potentialMate instanceof Male) {

//First check if any males are within matingrange

if (potentialMate.isAlive()) {

if (withinDistance(this, potentialMate) <= SwampNR.MATINGRANGE) {

this.myPlan.attemptMate = true;

this.myPlan.potentialMate = potentialMate.getTreeFrogState();

return;

} else {

livingMales.add(potentialMate.getTreeFrogState());

}

}

}

}

//Filter males based on whether they are calling

livingMales = filterByIfAudible(livingMales);

// No Mate in range

// Choose A Mate to approach

if (livingMales.size() > 0) {

myPlan.potentialMate = this.chooseMale(livingMales);

} else {

myPlan.potentialMate = null;

}

String chosenMaleStr = GetChosenMaleString();

if(!listOfChosenMales.isEmpty() && (!chosenMaleStr.equals(""))) {

if(!chosenMaleStr.equals(listOfChosenMales.get(listOfChosenMales.size()-1))) {

listOfChosenMales.add(chosenMaleStr);

}

}

else {

listOfChosenMales.add(chosenMaleStr);

}

}

/**

* Filter males based on whether they are calling

* @param potentialMales All males in the swamp

* @return Audible males in the swamp

*/

public ArrayList<TreeFrogState> filterByIfAudible(ArrayList<TreeFrogState> potentialMales) {

ArrayList<TreeFrogState> livingMales = new ArrayList<>();

for (TreeFrogState potentialMale : potentialMales) {

if (potentialMale.getPulseNumber() > 0) {

livingMales.add(potentialMale);

}

}

return livingMales;

}

/**

* Female leaps toward a previously chosen male

* @param maleList all males that the female can interact with physically

*/

public void act(Hashtable<String, TreeFrog> maleList) {

if (!getTreeFrogState().isAlive || this.getTreeFrogState().mated) {

return;

}

if (myPlan.attemptMate && myPlan.potentialMate != null) {

if (myPlan.potentialMate.isAlive && // someone else may have gotten

withinDistance(this, myPlan.potentialMate) <= SwampNR.MATINGRANGE) {

this.getTreeFrogState().potentialPartnerID = myPlan.potentialMate.getAgentID();

} else {

myPlan.attemptMate = false;

}

} else {

boolean updatedDistance = false;

boolean followingMale;

if (myPlan.potentialMate != null) {

double tempCoord = myPlan.potentialMate.getX();

if (headingX != tempCoord) {

headingX = tempCoord;

updateDistance();

updatedDistance = true;

}

tempCoord = myPlan.potentialMate.getY();

if (headingY != tempCoord) {

headingY = tempCoord;

if (!updatedDistance) {

updateDistance();

}

}

double tempY = Math.abs(headingY - getY());

double tempX = Math.abs(headingX - getX());

headingAngle = Math.atan(tempY / tempX);

followingMale = true;

getTreeFrogState().currentFrogToPursue = myPlan.potentialMate.getAgentID();

}

else {

// no male around to be chosen

getTreeFrogState().currentFrogToPursue = "none";

followingMale = false;

}

updatePosition(followingMale);

}

}

/**

* Set the strategy used by the female for pursuing a male

* @param mRule The strategy used by the female (random, best-of-n or min-thresh)

*/

public void setMatingRule(SwampNR.MatingRules mRule) {

matingRule = mRule;

}

/**

* Set the parameter for the best-of-n strategy

* @param nToMate Number of males to consider

*/

public void setNtoMate(int nToMate) {

NTOMATE = nToMate;

}

/**

* Set the parameter for the min-thresh strategy

* @param minTh The minimum pulses per call a male should have in order to be chosen

*/

public void setMinThreshold(int minTh) {

minThreshold = minTh;

}

/**

* When the frog is changing heading, it's the moment to update the distance

* it's travelled. Method called when it changes heading or mates

*/

private void updateDistance() {

if(!updatedSubSeq)

{

subseqX = getInitialX();

subseqY = getInitialY();

updatedSubSeq = true;

}

double tempX = subseqX - getX();

double tempY = subseqY - getY();

double distance = Math.sqrt(tempX * tempX + tempY * tempY);

distanceTravelled += distance;

subseqX = getX();

subseqY = getY();

}

/**

* Gives the original X position of the female

* @return X position of the female

*/

public double getInitialX() {

return initialX;

}

/**

* Sets the original X position of the female

* @param x position of the female

*/

public void setInitialX(double x) {

initialX = x;

}

/**

* Gives the original Y position of the female

* @return X position of the female

*/

public double getInitialY() {

return initialY;

}

/**

* Sets the original Y position of the female

* @param y position of the female

*/

public void setInitialY(double y) {

initialY = y;

}

/**

* Gives the distance travelled by a female

* @return distanceTravelled the distance travelled since the start of the simulation

*/

public double getDistanceTravelled() {

updateDistance();

return distanceTravelled;

}

/**

* Updates the position of the frog given the heading and current position

* @param phonotaxis true shows that the frog found a suitable male to mate,

* otherwise, it will start a random walk

*/

private void updatePosition(boolean phonotaxis) {

double tempX = headingX - getX();

double tempY = headingY - getY();

int negX = -1;

int negY = -1;

if (tempX > 0) {

negX = 1;

}

if (tempY > 0) {

negY = 1;

}

if(randomNegX == 0 && randomNegY == 0)

{

if(random.nextBoolean())

randomNegX = 1;

else

randomNegX = -1;

if(random.nextBoolean())

randomNegY = 1;

else

randomNegY = -1;

}

double tX, tY;

if (!phonotaxis) {

tX = getX() + randomNegX * speedRandom * Math.cos(headingAngle);

tY = getY() + randomNegY * speedRandom * Math.sin(headingAngle);

} else {

tX = getX() + negX * speedPhonotaxis * Math.cos(headingAngle);

tY = getY() + negY * speedPhonotaxis * Math.sin(headingAngle);

}

if (tX > SwampDimensions.getSwampWidth()) {

tX = SwampDimensions.getSwampWidth();

}

else if(tX < 0.0) {

tX = 0.0;

}

if (tY > SwampDimensions.getSwampHeight()) {

tY = SwampDimensions.getSwampHeight();

}

else if(tY < 0.0) {

tY = 0.0;

}

setX(tX);

setY(tY);

}

/**

* This method is the implementation of the choice strategy it invokes the

* current strategy that the females are using to choose the male to mate.

* @param possibleMales All males in the swamp

* @return Chosen male that the female will leap toward

*/

private TreeFrogState chooseMale(ArrayList<TreeFrogState> possibleMales) {

TreeFrogState chosen = null;

switch (matingRule) {

case bestOfN:

chosen = bestOfNnearest(possibleMales, NTOMATE);

break;

case minThreshold:

chosen = minThreshold(possibleMales);

break;

case random:

chosen = randomPartner(possibleMales);

break;

default:

System.err.println("UNRECOGNIZED MATING RULE " + matingRule);

break;

}

return chosen;

}

/**

* Chooses the best male of those N nearest. The method

* first gets the nearest n males into the toChoose ArrayList

* then, it returns the best male

* @param possibleMales All males in the swamp

* @param n Number of closest males

* @return Chosen male that the female will leap toward

*/

private TreeFrogState bestOfNnearest(

ArrayList<TreeFrogState> possibleMales, int n) {

int males = possibleMales.size();

TreeFrogState[] toChoose = new TreeFrogState[n];

double distance[] = new double[n]; // to keep the distances, so the

// calculation

// is faster for checking the males

int pulsesPerCall[] = new int[n];

for (int j = 0; j < n; j++) {

distance[j] = 1000000;

}

for (int i = 0; i < males; i++) {

TreeFrogState currentMale = possibleMales.get(i);

double thisMaleDistance = distanceToFrog(currentMale);

try {

if (i > n - 1) {

// first fill in the N males, then reduce the distances

sortMalesByDistance(distance, toChoose, pulsesPerCall);

if (thisMaleDistance < distance[0]) {

distance[0] = thisMaleDistance;

toChoose[0] = currentMale;

pulsesPerCall[0] = currentMale.getPulseNumber();

}

} else {

distance[i] = thisMaleDistance;

toChoose[i] = currentMale;

pulsesPerCall[i] = currentMale.getPulseNumber();

}

} catch (Exception e) {

System.out.println(e);

}

}

// Now that the nearest males are in the possibleMales pick the best

// caller

TreeFrogState bestMale;

int bestPulse = pulsesPerCall[0];

int best = 0;

for (int i = 1; i < n; i++) {

int currentPulse = pulsesPerCall[i];

if (bestPulse < currentPulse) {

best = i;

bestPulse = currentPulse;

} else // if there are 2 callers with the same pulse number

// choose nearest one

if (bestPulse == currentPulse) {

if (distance[i] < distance[best]) {

best = i;

}

}

}

bestMale = toChoose[best];

return bestMale;

}

/**

* Sorts the males before the others are checked, so the greater the

* distance, the closer to 0.

* @param distance Array of distances

* @param toSort Array of frogs

* @param pulses Array of pulses per call

*/

private void sortMalesByDistance(double distance[], TreeFrogState[] toSort,

int[] pulses) {

int dist = distance.length;

try {

for (int i = 0; i < dist - 1; i++) {

for (int j = 1; j < dist; j++) {

if (distance[j - 1] < distance[j]) {

double temp = distance[j - 1];

distance[j - 1] = distance[j];

distance[j] = temp;

TreeFrogState tempMale = toSort[j - 1];

toSort[j - 1] = toSort[j];

toSort[j] = tempMale;

int tempPulse = pulses[j - 1];

pulses[j - 1] = pulses[j];

pulses[j] = tempPulse;

}

}

}

} catch (Exception e) {

System.out.println(e.toString());

}

}

/**

* Takes the males above the min. threshold and picks the closest to the female

* @param possibleMales All males in the swamp

* @return Chosen male that the female will leap toward

*/

private TreeFrogState minThreshold(ArrayList<TreeFrogState> possibleMales) {

TreeFrogState best = null;

ArrayList<TreeFrogState> toMate;

if (!possibleMales.isEmpty()) {

toMate = getMinThresholdList(possibleMales);

if (!toMate.isEmpty()) {

// start with the first in the list and then see if there are

// any closer ones

best = toMate.get(0);

double currentNearest = withinDistance(toMate.get(0), this.getTreeFrogState());

for (int i = 1; i < toMate.size(); i++) {

TreeFrogState temp = toMate.get(i);

double currentDist = withinDistance(temp, this.getTreeFrogState());

if (currentDist < currentNearest) {

best = temp;

currentNearest = currentDist;

}

}

}

}

return best;

}

/**

* Get all males that have a higher pulses per call than the threshold

* @param possibleMales All males in the swamp

* @return List of males that have a higher value of pulses per call than the threshold

*/

private ArrayList<TreeFrogState> getMinThresholdList(ArrayList<TreeFrogState> possibleMales) {

ArrayList<TreeFrogState> toMate = new ArrayList<>();

if (!possibleMales.isEmpty()) {

for (TreeFrogState temp : possibleMales) {

if (temp.getPulseNumber()>= minThreshold) {

toMate.add(temp);

}

}

}

return toMate;

}

/**

* Pick a random partner in the swamp

* @param possibleMales All males in the swamp

* @return Chosen male that the female will leap toward

*/

private TreeFrogState randomPartner(ArrayList<TreeFrogState> possibleMales) {

TreeFrogState temp = null;

if (chosenBefore == null || !chosenBefore.isAlive) {

while (temp == null) {

try {

// MS: made this a local random choice

double tmp = random.nextDouble();

int picked = (int) (tmp * possibleMales.size());

temp = possibleMales.get(picked);

} catch (Exception e) {

temp = null;

}

chosenBefore = temp;

}

}

//If a new male was chosen, then return, otherwise, return the last chosen

temp = chosenBefore;

return temp;

}

/**

* Remove the frog from the simulation

* @param reason The reason why the frog was removed (e.g., she mated)

*/

@Override

public void die(String reason) {

getTreeFrogState().isAlive = false;

deathReason = reason;

updateDistance();

}

/**

* Return an string containing the chosen male and its pulse number

* @return String on the format ID*PN

*/

private String GetChosenMaleString() {

if(myPlan.potentialMate != null)

{

return myPlan.potentialMate.getAgentID() + "*" + myPlan.potentialMate.getPulseNumber();

}

return "";

}

/**

* Return the list of males chosen by the female

* @return ArrayList with chosen males

*/

public ArrayList<String> GetChosenMaleListString() {

return listOfChosenMales;

}

}

/**

* Wrapper for a male frog

*/

class Male extends TreeFrog {

double initialHeading = random.nextDouble() * 360;

/**

* Creates the frog with position and calculates his pulses per call

* @param agentID ID of the frog

* @param iX X Position of the frog

* @param iY Y Position of the frog

* @param seed Random seed

* @param meanCall The mean of pulses per call

* @param stdCall Standard deviation of pulses per call

*/

public Male(String agentID, double iX, double iY, long seed, int meanCall, int stdCall) {

super(agentID, iX, iY, seed);

setGaussianPulseNumber(meanCall, stdCall);

size = 4.72;

}

/**

* Creates the frog with specific position and pulses per call

* @param agentID ID of the frog

* @param iX X Position of the frog

* @param iY Y Position of the frog

* @param seed Random seed

* @param pulseNumber Pulse number for the male

*/

public Male(String agentID, double iX, double iY, long seed, int pulseNumber) {

super(agentID, iX, iY, seed);

setPulseNumber(pulseNumber);

size = 4.72; // default size for males

}

/**

* Calculates the pulse number

* @param meanCall Mean value for the pulses

* @param stdCall Standard deviation for the pulses

*/

private void setGaussianPulseNumber(int meanCall, int stdCall) {

int pulseNumber = (int) (0.5 + random.nextGaussian(meanCall, stdCall));

while (pulseNumber < SwampNR.minPulseNumber

|| pulseNumber > SwampNR.maxPulseNumber) {

pulseNumber = (int) (0.5 + random.nextGaussian(meanCall, stdCall));

}

setPulseNumber(pulseNumber);

}

/**

* Gets the pulse number for this male

* @return int containing the pulse number

*/

public int getPulseNumber() {

return getTreeFrogState().getPulseNumber();

}

/**

* Sets the pulse number for this male

* @param pn The value of the pulse number

*/

public final void setPulseNumber(int pn) {

getTreeFrogState().setPulseNumber(pn);

}

/**

* Print the male in a standardized way

* @return string with the cycle number, id, position and pulses per call of the male

*/

@Override

public String toString() {

String ret = getTreeFrogState().getAgentID() + "," + getX() + "," + getY()

+ "," + getPulseNumber();

return ret;

}

/**

* Male leaps toward a previously chosen male or just call. Also verify if two frogs have mated.

* @param femaleList all females that the male can interact with physically

*/

public void act(Hashtable<String,TreeFrog> femaleList) {

TreeFrogState tfs = getTreeFrogState();

if (!isAlive() || tfs.mated) {

return;

}

for (TreeFrog potentialMate : femaleList.values()) {

if(potentialMate instanceof Female) {

if (potentialMate.isAlive()) {

if ((withinDistance(this, potentialMate) <= SwampNR.MATINGRANGE)) {

TreeFrogState pmstate = potentialMate.getTreeFrogState();

if (pmstate.potentialPartnerID.compareTo(tfs.getAgentID()) == 0) {

tfs.lastMateID = potentialMate.getTreeFrogState().getAgentID();

tfs.mated = true;

SwampNR.reportMating(getTreeFrogState().getAgentID(), tfs.lastMateID, getTreeFrogState().getPulseNumber());

this.die("mate");

return;

}

}

}

}

}

}

/**

* Remove the frog from the simulation

* @param reason The reason why the frog was removed (i.e., he mated)

*/

@Override

public void die(String reason) {

if (reason.compareTo("mate") == 0) {

getTreeFrogState().isAlive = false;

}

deathReason = reason;

}

}

/**

* Abstract class to define a frog

*/

abstract class TreeFrog {

//Store the state variables of the frog

private TreeFrogState agentState;

//Seed to generate random numbers for the frog

private double seed = 0.0;

//Random number generator

public SpecialRandom random;

//Size of a frog

protected double size = 4.72;//cms

//Reason of removing the frog from the simulation

protected String deathReason = "none";

// needs to be implemented by each agent

abstract void die(String dC);

abstract void act(Hashtable<String, TreeFrog> localworld);

/**

* Creates the frog in the given position Max

* @param agentID ID of the frog

* @param X X Position of the frog

* @param Y Y Position of the frog

* @param seed Random seed

*/

public TreeFrog(String agentID, double X, double Y, long seed) {

agentState = new TreeFrogState(agentID, X, Y);

this.seed = seed;

random = new SpecialRandom(seed); // seed the local random number generator

}

/**

* Get data from the tree frog

* @return A TreeFrogState with data from the agent

*/

public TreeFrogState getTreeFrogState() {

return agentState;

}

/**

* Get the X position of the frog

* @return double with the X position of the frog

*/

public double getX() {

return getTreeFrogState().getX();

}

/**

* Get the Y position of the frog

* @return double with the Y position of the frog

*/

public double getY() {

return getTreeFrogState().getY();

}

/**

* Set the X position of the frog

* @param double with the X position of the frog

*/

public void setX(double X) {

getTreeFrogState().setX(X);

}

/**

* Set the Y position of the frog

* @param double with the Y position of the frog

*/

public void setY(double Y) {

getTreeFrogState().setY(Y);

}

/**

* Evaluate the fitness of the male. For now, it just the pulse number

* @param male Frog to being evaluated

* @return The pulse number of the male

*/

public double evaluateMale(TreeFrogState male) {

return (male.getPulseNumber());

}

/**

* Standard string containing the frog position and status

* @return String with the data from the frog

*/

@Override

public String toString() {

String temp = "" + getX() + "," + getY() + " " + isAlive() + " " + deathReason;

return temp;

}

/**

* Verify if the frog is alive

* @return true if the frog can still mate

*/

public boolean isAlive() {

return getTreeFrogState().isAlive;

}

/**

* Calculates the Euclidian distance between 2 frogs

* @param frog First frog

* @param frog2 Second frog

* @return double containing the euclidian distance

*/

static public double withinDistance(TreeFrog frog, TreeFrog frog2) {

double frogsX = frog.getX() - frog2.getX();

double frogsY = frog.getY() - frog2.getY();

return Math.sqrt(frogsX * frogsX + frogsY * frogsY);

}

/**

* Calculates the Euclidian distance between 2 frogs

* @param frog First frog

* @param frog2 Second frog

* @return double containing the euclidian distance

*/

static public double withinDistance(TreeFrog frog, TreeFrogState frog2) {

double frogsX = frog.getX() - frog2.getX();

double frogsY = frog.getY() - frog2.getY();

return Math.sqrt(frogsX * frogsX + frogsY * frogsY);

}

/**

* Calculates the Euclidian distance between 2 frogs

* @param frog First frog

* @param frog2 Second frog

* @return double containing the euclidian distance

*/

static public double withinDistance(TreeFrogState frog, TreeFrogState frog2) {

double frogsX = frog.getX() - frog2.getX();

double frogsY = frog.getY() - frog2.getY();

return Math.sqrt(frogsX * frogsX + frogsY * frogsY);

}

/**

* Calculates the current male's distance to a frog

* @param toCheck Male which the distance should be calculated

* @return double containing the euclidian distance

*/

public double distanceToFrog(Male toCheck) {

double maleX = toCheck.getX();

double maleY = toCheck.getY();

double difX = getX() - maleX;

double difY = getY() - maleY;

return Math.sqrt(difX * difX + difY * difY);

}

/**

* Calculates the current male's distance to an agent

* @param toCheck Agent which the distance should be calculated

* @return double containing the euclidian distance

*/

public double distanceToFrog(TreeFrogState toCheck) {

double maleX = toCheck.getX();

double maleY = toCheck.getY();

double difX = getX() - maleX;

double difY = getY() - maleY;

double difZ = 0;

return Math.sqrt(difX * difX + difY * difY + difZ * difZ);

}

}

/**

* Encapsulates the data of the treefrog

*/

class TreeFrogState {

//ID of a frog that can be a mate

public String potentialPartnerID = "";//coordinated mating decision

//ID of the partner

public String lastMateID = "";

//Frog have mated

public boolean mated = false;

//Contains the id of the frog chosen by the female

public String currentFrogToPursue = "";

//Pulse number of male frogs

public int pulseNumber = 0;

//Id of the frog

private String agentID;

//Position of the frog in the swamp

public double X = 0;

public double Y = 0;

//Frog is still sensing and acting

public boolean isAlive = true;

public boolean alive = true;

/**

* Creates the frog in a specific position

* @param agentID ID of the frog

* @param x X Position of the frog

* @param y Y Position of the frog

*/

public TreeFrogState(String agentID, double x, double y) {

this.agentID = agentID;

this.X = x;

this.Y = y;

}

/**

* Get the pulse number of the frog

* @return int containing the pulse number

*/

public int getPulseNumber() {

return pulseNumber;

}

/**

* Set the pulse number of the frog

* @param x int containing the pulse number

*/

public void setPulseNumber(int x) {

pulseNumber = x;

}

/**

* Get the id of the agent

* @return String with the ID format: sex+number

*/

public String getAgentID() {

return agentID;

}

/**

* Get the x position of the agent

* @return double containing the x position

*/

public double getX() {

return X;

}

/**

* Get the y position of the agent

* @return double containing the y position

*/

public double getY() {

return Y;

}

/**

* Set the x position of the agent

* @param x x position

*/

public void setX(double x) {

X = x;

}

/**

* Set the y position of the agent

* @param y y position

*/

public void setY(double y) {

Y = y;

}

/**

* Print the frog in a standardized way

* @return string with the frog's parameters

*/

@Override

public String toString() {

return super.toString() + "##TreeFrogState##" + "isAlive:" + isAlive + ":mated:" + mated + ":currentFrogToPursue:" + currentFrogToPursue + ":pulseNumber:" + pulseNumber + ":potentialPartnerID:" + potentialPartnerID + ":lastMateID:" + lastMateID;

}

}

/**

* Encapsulates the dimensions of the swamp

*/

class SwampDimensions {

private static int SWAMPWIDTH = 0;

private static int SWAMPHEIGHT = 0;

/**

* Set the dimensions of the swamp

* @param swampWidth int containing the width of the swamp

* @param swampHeight int containing the height of the swamp

*/

public static void setSwampDimensions(int swampWidth, int swampHeight) {

SWAMPWIDTH = swampWidth;

SWAMPHEIGHT = swampHeight;

}

/**

* Get the swamp width

* @return int containing the width of the swamp

*/

public static int getSwampWidth() {

return SWAMPWIDTH;

}

/**

* Get the swamp height

* @return int containing the height of the swamp

*/

public static int getSwampHeight() {

return SWAMPHEIGHT;

}

}

/**

* ArrayList of females

*/

class FemaleArrayList

{

private ArrayList<Female> femaleArray;

/**

* Creates a female array list

* @param array An array of existent females in the swamp

*/

public FemaleArrayList(ArrayList<Female> array) {

femaleArray = array;

}

/**

* Print the array of females in a standardized way

*/

@Override

public String toString() {

String returnString = "";

int listSize = femaleArray.size();

for (int t = 0; t < listSize; ++t) {

Female frog = femaleArray.get(t);

ArrayList<String> chosenMaleList = frog.GetChosenMaleListString();

if(t == 0) {

returnString += frog.getTreeFrogState().getAgentID() + "[" + "(" + frog.getInitialX() + "#" + frog.getInitialY() + "):" + "(" + frog.getX() + "#" + frog.getY() + ")" + "]";

}

else {

returnString += ";" + frog.getTreeFrogState().getAgentID() + "[" + "(" + frog.getInitialX() + "#" + frog.getInitialY() + "):" + "(" + frog.getX() + "#" + frog.getY() + ")" + "]";

}

returnString += AddChosenMaleListStr(chosenMaleList);

}

return returnString;

}

/**

* Add a list of strings identifying the chosen males (ID + PN)

* @param chosenMaleList ArrayList of chosen males

* @return A formatted string

*/

private String AddChosenMaleListStr(ArrayList<String> chosenMaleList) {

String returnString = "";

for (int i=0; i<chosenMaleList.size(); ++i) {

if(i==0) {

returnString += "{" + chosenMaleList.get(i);

}

else {

returnString += "|" + chosenMaleList.get(i);

}

}

returnString += "}";

return returnString;

}

/**

* Get the array of females

* @return The array of females

*/

public ArrayList<Female> get() {

return femaleArray;

}

}

/**

* ArrayList of males

*/

class MaleArrayList

{

private ArrayList<Male> maleArray;

/**

* Creates a male array list

* @param array An array of existent males in the swamp

*/

public MaleArrayList(ArrayList<Male> array) {

maleArray = array;

}

/**

* Print the array of males in a standardized way

*/

@Override

public String toString() {

String returnString = "";

int listSize = maleArray.size();

for (int t = 0; t < listSize; ++t) {

Male frog = maleArray.get(t);

if(t == 0)

returnString += frog.getTreeFrogState().getAgentID() + "[" + "(" + frog.getX() + "#" + frog.getY() + ")" + "]";

else

returnString += ";" + frog.getTreeFrogState().getAgentID() + "[" + "(" + frog.getX() + "#" + frog.getY() + ")" + "]";

}

return returnString;

}

/**

* Get the array of males

* @return The array of males

*/

public ArrayList<Male> get() {

return maleArray;

}

}

/**

* New type of random number generator

* created to generate numbers in a bimodal (inversegaussian)

* distribution

*/

class SpecialRandom extends Random {

long seedUsed;

//----------------------------------------------

String[] seedName;

SpecialRandom[] seedValue;

int agentCount = 0;

//----------------------------------------------

public static final long serialVersionUID = 1;

public SpecialRandom(long seed) {

super(seed);

seedUsed = seed;

}

//----------------------------------------------

public SpecialRandom(long seed, int size, String[] classNames) {

super(seed);

seedUsed = seed;

seedName = classNames;

seedValue = new SpecialRandom[size];

for (int i = 0; i < size; i++) {

seedValue[i] = new SpecialRandom(seed); //Does each need to be unique?? seedValue[i] = new SpecialRandom(seed+i);

System.out.print(seedName[i] + ": " + seedValue[i].seedUsed);

}

}

public double nextDouble(Object obj) {

for (int i = 0; i < seedName.length; i++) {

if (obj.getClass().getName().equals("com.grid.simulations.simworld." +seedName[i])) {

agentCount++;

return (seedValue[i].nextDouble());

}

}

return (((long) next(26) << 27) + next(27))

/ (double) (1L << 53);

}

public long nextLong(Object obj) {

for (int i = 0; i < seedName.length; i++) {

if (obj.getClass().getName().equals("com.grid.simulations.simworld." + seedName[i])) {

agentCount++;

return (seedValue[i].nextLong());

}

}

System.out.print("class not found!");

return (((long) next(26) << 27) + next(27))

/ (long) (1L << 53);

}

public long nextLong(Class obj) {

for (int i = 0; i < seedName.length; i++) {

if (obj.getName().equals("com.grid.simulations.simworld." + seedName[i])) {

agentCount++;

return (seedValue[i].nextLong());

}

}

System.out.print("class not found!");

return (((long) next(26) << 27) + next(27))

/ (long) (1L << 53);

}

//----------------------------------------------

/**

* Returns the next Gaussian sampled to the

* mean and standard deviation required

* */

public double nextGaussian(double mean, double std) {

return mean + std * nextGaussian();

}

/**

* Returns the next random number in the bimodal

* m1 and m2 are the means, std the standard deviation

*/

public double nextBimodal(double m1, double m2, double std) {

double temp = nextDouble();

if (nextDouble() <= 0.5) {

temp = m1 - (temp * std);

} else {

temp = m2 + (temp * std);

}

return temp;

}

public long getSeedUsed() {

return seedUsed;

}

public void setSeedUsed(long seedUsed) {

this.seedUsed = seedUsed;

}

}
