# Supplementary figures and images for: Mate choice strategies in a spatially-explicit model environment
Source: PLoS One. 2018 Aug 23;13(8):e0202680. doi: 10.1371/journal.pone.0202680 (PMC6107201; doi:10.1371/journal.pone.0202680)

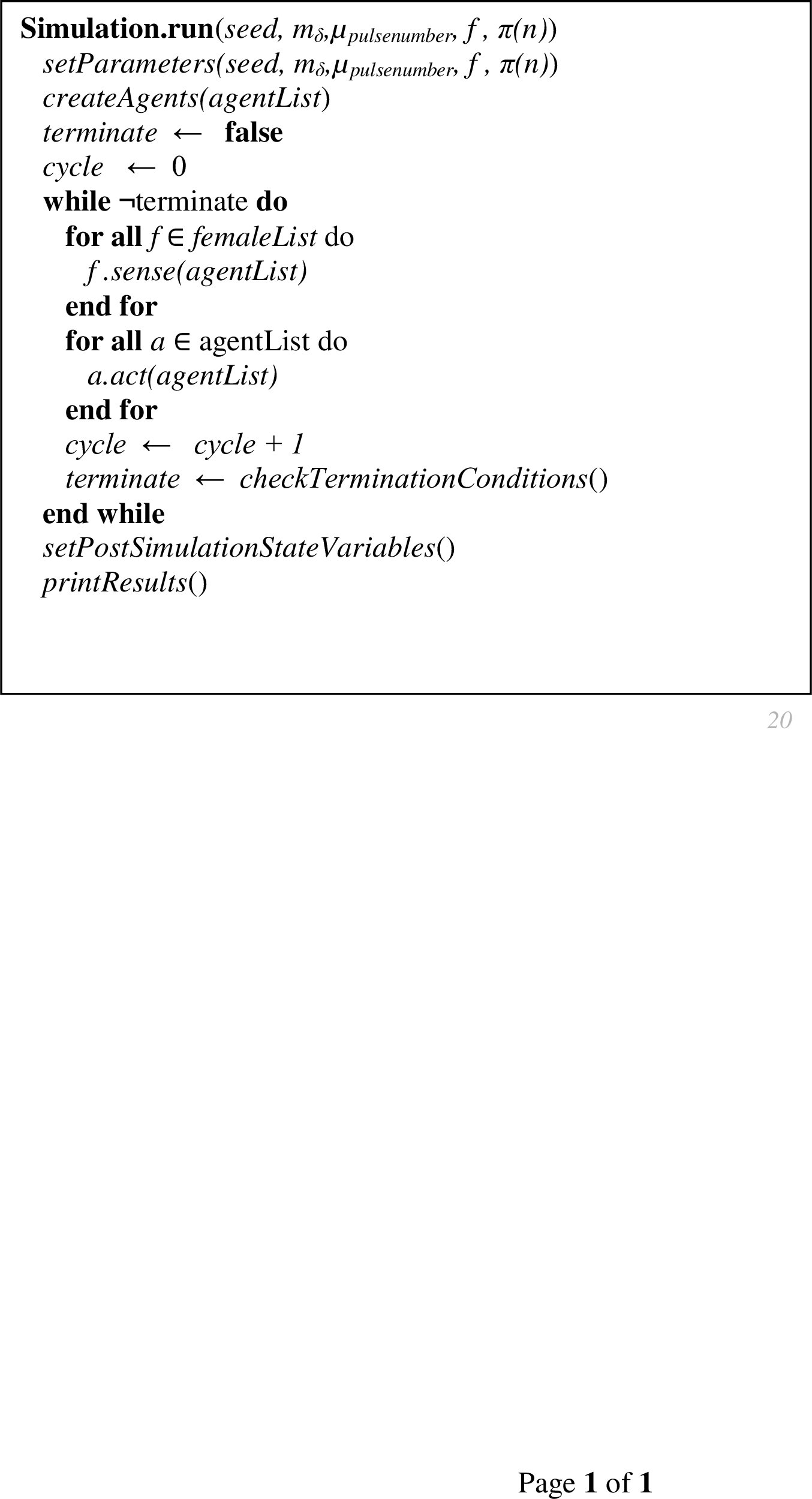

Supplement: S1 Fig — (TIF) [file pone.0202680.s002.tif]

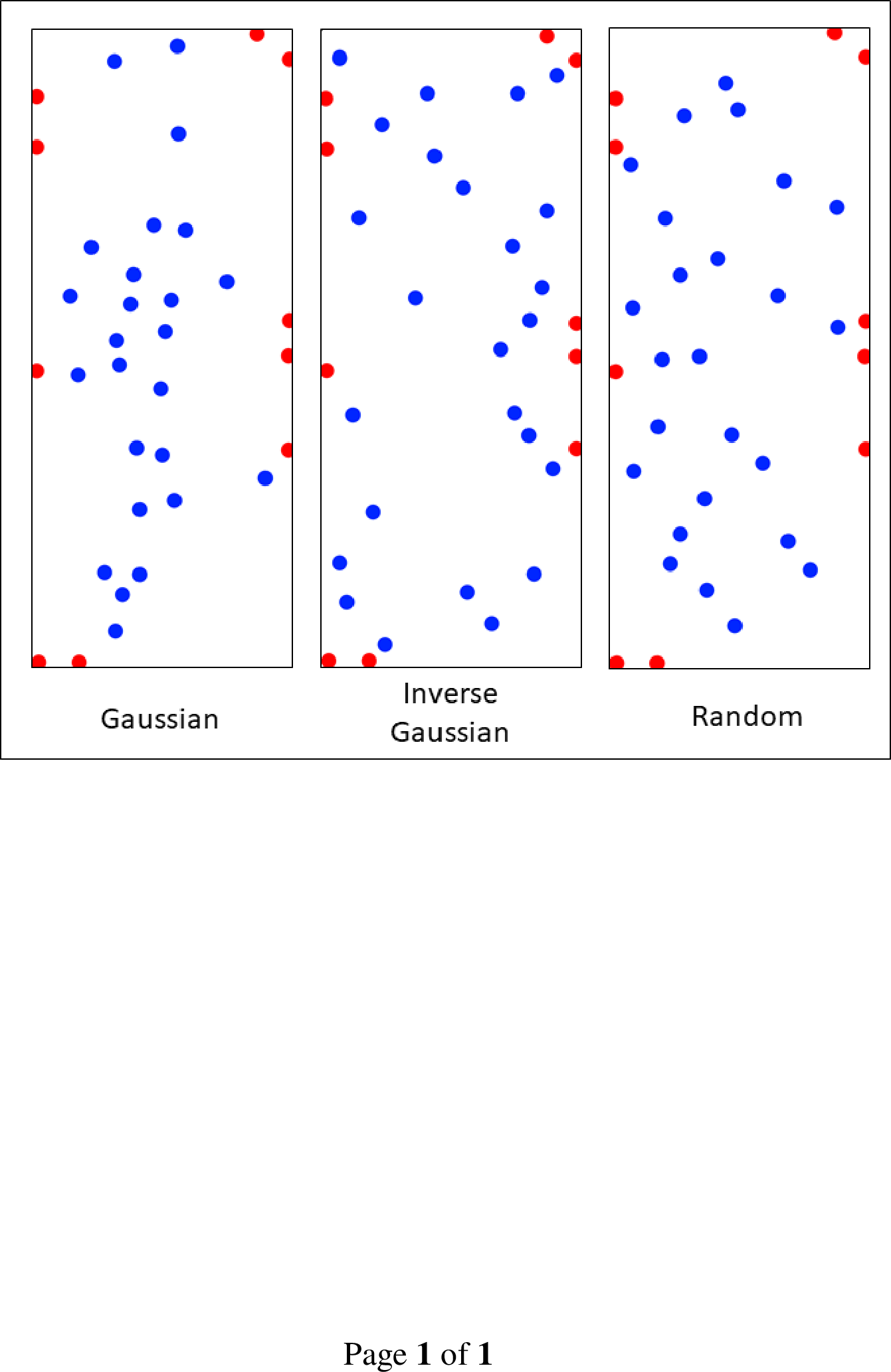

Supplement: S2 Fig — Locations of males are shown in blue and females in red. The dot size is not to scale but has been increased for visualization purposes. (TIF) [file pone.0202680.s003.tif]
